# A novel signature constructed by ferroptosis-associated genes (FAGs) for the prediction of prognosis in bladder urothelial carcinoma (BLCA) and associated with immune infiltration

**DOI:** 10.1186/s12935-021-02096-3

**Published:** 2021-08-06

**Authors:** Jiao-chen Luan, Teng-yue Zeng, Qi-jie Zhang, De-run Xia, Rong Cong, Liang-yu Yao, Le-bin Song, Xiang Zhou, Xuan Zhou, Xiang Chen, Jia-dong Xia, Ning-hong Song

**Affiliations:** 1 Department of Urology, The First Affiliated Hospital of Nanjing Medical University, 300 Guangzhou Road, Nanjing, China; 2 Department of Epidemiology and Biostatistics, School of Public Health, Nanjing Medical University, Nanjing, 211166 Jiangsu China; 3 Department of Dermatology, The First Affiliated Hospital of Nanjing Medical University, Nanjing, China; 4 Key Laboratory of Cardiovascular and Cerebrovascular Medicine, School of Pharmacy, Nanjing Medical University, Nanjing, China; 5 The Affiliated Kezhou People’s Hospital of Nanjing Medical University, Kezhou, Xinjiang China

**Keywords:** Bladder cancer, Ferroptosis, Prognosis, Immune infiltration, TFRC

## Abstract

**Background:**

Ferroptosis, a novel form of regulated cell death, has been implicated in the pathogenesis of cancers. Nevertheless, the potential function and prognostic values of ferroptosis in bladder urothelial carcinoma (BLCA) are complex and remain to be clarified. Therefore, we proposed to systematically examine the roles of ferroptosis-associated genes (FAGs) in BLCA.

**Methods:**

According to The Cancer Genome Atlas (TCGA) database, differently expressed FAGs (DEFAGs) and differently expressed transcription factors (DETFs) were identified in BLCA. Next, the network between DEFAGs and DETFs, GO annotations and KEGG pathway analyses were performed. Then, through univariate, LASSO and multivariate regression analyses, a novel signature based on FAGs was constructed. Moreover, survival analysis, PCA analysis, t-SNE analysis, ROC analysis, independent prognostic analysis, clinicopathological and immune correlation analysis, and experimental validation were utilized to evaluate the signature.

**Results:**

Twenty-eight DEFAGs were identified, and four FAGs (CRYAB, TFRC, SQLE and G6PD) were finally utilized to establish the FAGs based signature in the TCGA cohort, which was subsequently validated in the GEO database. Moreover, we found that immune cell infiltration, immunotherapy-related biomarkers and immune-related pathways were significantly different between two risk groups. Besides, nine molecule drugs with the potential to treat bladder cancer were identified by the connectivity map database analysis. Finally, the expression levels of crucial FAGs were verified by the experiment, which were consistent with our bioinformatics analysis, and knockdown of TFRC could inhibit cell proliferation and colony formation in BLCA cell lines in vitro.

**Conclusions:**

Our study identified prognostic ferroptosis-associated genes and established a novel FAGs signature, which could accurately predict prognosis in BLCA patients.

**Supplementary Information:**

The online version contains supplementary material available at 10.1186/s12935-021-02096-3.

## Introduction

Bladder cancer (BLCA), one of the most common malignant and highly aggressive tumors worldwide, was reported to lead to an estimated 81,190 newly diagnosed cases and 17,240 deaths in the United States [1]. Based on the pathological diagnosis, BLCA can be generally categorized into muscle invasive bladder cancer (MIBC) and non-muscle invasive bladder cancer (NMIBC) [2, 3]. Regarding the management of non-muscle invasive bladder cancer (NMIBC), transurethral resection of the bladder (TURB) is the most preferred therapy [3]. For muscle invasive bladder cancer (MIBC), neoadjuvant chemotherapies utilizing a cisplatin combination regimen plus with radical cystectomy are preferred [4, 5]. For BLCA patients with advanced metastasis, chemotherapy may be considered as the mainstream treatment [4, 5]. Recently, various types of immunotherapy have been applied to treat tumors, such as immune checkpoint inhibitors (ICIs), vaccines, monoclonal antibodies, T‐cell transfer therapies and immune system modulators [6]. Immunotherapy in tumor management is the method which can involve the immune system of patients to increase or modify the defense mechanism against the developing tumor [7]. Notably, BLCA is identified to be immunogenic and is responsive to immunotherapy, including ICIs and intravesical Bacillus Calmette–Guerin (BCG). intravesical induction BCG immunotherapy was recommended as the guideline for patients with an intermediate or high-risk non-muscle invasive bladder cancer (NMIBC) [8, 9]. With respect to ICIs, a number of trials have explored the roles of ICIs in advanced BLCA, such as avelumab, atezolizumab, nivolumab and durvalumab. Although different kinds of trials reveal the promising outcomes and improvements, less than half of advanced BLCA patients benefit from ICIs therapy [10]. Due to its high recurrence and poor prognosis, BLCA was considered as an enormous threat to human health [11, 12]. Therefore, it is of great significance to identify the effective prognosis prediction model for patients in the whole course of the disease.

Ferroptosis, serving as the novel form of regulated cell death, is distinct from necrosis, apoptosis and autophagy at morphological, biochemical and genetical levels. It is characterized by lipid reactive oxygen species accumulation and lipid peroxidation reaching lethal levels [13–15]. Recently, increasing researches has demonstrated that ferroptosis is relevant to the process of tumor genesis and progression, and inducing ferroptosis is thought as a potential tumor treatment that can selectively eliminate some cancer cells [13, 16, 17]. For example, GPX4 is believed to be the main enzyme preventing ferroptosis, and the induction of ferroptosis by suppressing GPX4 has been a therapeutic method for tumor cell death [18–20]. TFRCs took part in sulfasalazine-induced ferroptosis in breast cancer [21]. Several studies revealed that p53 could suppress tumor development by regulating FAGs to inhibit tumor development, and GLS2, PTGS2, SAT1 and SLC7A11 have been demonstrated to serve as target genes for ferroptosis [13]. However, whether the ferroptosis-associated genes (FAGs) are significantly related to the prognosis of Bladder urothelial cancer patients remains unknown.

In our study, we first obtained mRNA expression profiles and clinicopathological parameters of BLCA patients from the Cancer Genome Atlas (TCGA) and the Gene Expression Omnibus (GEO). Then, pathway enrichment and functional annotation analysis was performed to investigate the potential mechanisms of BLCA. Next, in accordance with the TCGA dataset, we established a prognostic signature based on ferroptosis-associated genes (FAGs), which was subsequently verified in another database (GSE13507). Moreover, we further discussed the association with the FAGs signature and immune cell infiltration, immune-related biomarkers and pathways. Additionally, the crucial gene TFRC was selected for experimental verification.

## Materials and methods

### Data collection

In our study, RNA-seq data of BLCA and normal bladder tissues with related clinical information were collected from the TCGA website (https://portal.gdc.cancer.gov/) and GEO (https://www.ncbi.nlm.nih.gov/geo/). The TCGA microarray was employed as the discovery set, and further was utilized to construct the signature that was systematically identified in the validation set (GSE13507 microarray). Additionally, sixty ferroptosis-associated genes (FAGs) were acquired from the known literature (Additional file 11: Table S1) [19, 20, 22, 23].

### Establishment and verification of the prognostic FAGs signature

Differently expressed FAGs (DEFAGs) between BLCA and normal solid tissue samples were identified by the “limma” R package in the TCGA dataset. The univariate Cox was applied to investigate the prognostic value of DEFAGs. LASSO Cox regression analysis was then applied to minimize the risk of overfitting, contributing to variable selection and regularization [24, 25]. Eventually, we established a prognostic model by employing the multiple stepwise Cox regression. The algorithm of each BLCA patient was constructed as follows:

$${\text{RiskScore}} = \sum\limits_{{{\text{i}} = 1}}^{{\text{n}}} {{\text{expi}}} \times \beta {\text{i}}$$

where, expi is the expression value of each candidate FAG, and βi is the regression coefficient of FAG i. Next, BLCA patients were stratified into high- and low-risk groups based on the median value of the riskScore for the following analysis.

### Functional enrichment analysis, PPI network construction and genetic alterations

The “clusterProfiler” R package was utilized to perform Gene ontology (GO) enrichment Analysis and Kyoto encyclopedia of genes and genomes (KEGG) pathway. The activity of thirteen immune-related pathways was calculated by performing single-sample gene set enrichment analysis (ssGSEA) in the “gsva” R package [26]. To further screen the interactive relationships between FAGs, DEFAGs were uploaded to the STRING (http://www.string-db.org/) dataset, and then by using Cytoscape software, PPI network was established. The genetic alterations of hub FAGs were obtained from the cBioPortal.

### Identification of a network between differently expressed transcription factors (DETFs) and FAGs

The Cistrome database (http://www.cistrome.org/) was applied to predict transcription factors targets in tumors. By utilizing “Lima” package in R software, DETFs were identified between normal samples and BLCA. Correlation test between DETFs and DEFAGs was performed. P value less than 0.01and correlation coefficient at least 0.4 were selected as the best correlated.

### Construction of the hybrid nomogram

Incorporated with the clinical parameters and the FAGs signature, a hybrid nomogram was constructed that could predict BLCA patients’ overall survival (1-, 3- and 5-year OS). The novel nomogram was verified by utilizing the calibration curve.

### Exploration of immunotherapy related biomarkers and tumor-infiltrating immune cells

The currently acknowledged methods, including XCELL [27, 28], TIMER [29, 30], QUANTISEQ [31, 32], MCPCOUNT [33], EPIC [34], CIBERSORT [30, 35] and CIBERSORT-ABS [36] were applied to measure the immune infiltration scores. Differences between two risk groups were analyzed by the Wilcoxon signed-rank test. The value of aneuploidy score and T cell receptor (TCR) richness were downloaded from Thorsson et al. [37]. Cytolytic activity (CYT) was calculated based on the geometric mean of PRF1 and GZMA (two cytolytic markers) [38]. Thirty-four immune checkpoint genes were obtained from Auslander et al. [39].

### Identification of candidate small molecule drugs

The Connectivity map (cMap) was utilized to facilitate researchers to predict potential drugs that might reverse or induce the biological states of diseases, and discover its possible mechanism. Differently expressed FAGs related to BLCA were uploaded to the CMAP in the “query” module. Next, functional connection between bioactive chemicals and FAGs was investigated.

### Cell culture and transfection

All cell lines were purchased from the Type Culture Collection of the Chinese Academy of Sciences (Shanghai, China). Two BLCA cell lines (J82 and UMUC3) were cultivated in DMEM (Gibco) containing 10% fetal bovine serum, and one normal human urothelial cell line (SV-HUC1) were grown in SV-HUC-1 cell special medium (procell, Wuhan, China). Cultures were incubated at 37 °C with 5% CO_2_ in a humidified atmosphere. The TFRC si-RNA was designed and synthesized by Hanbio (Shanghai, China). Lipofectamine 2000 (Invitrogen, USA) was utilized as a transfection reagent according to the instructions. J82 and UMUC3 cell lines were transfected with siRNA (10 nmol/l) utilizing Lipofectamine 2000 (5 μl per well, Invitrogen, USA) according to the manufacturer's protocol.

### RNA isolation and quantitative real-time PCR (qRT-PCR)

Total RNA was isolated from cells using TRIzol reagent (Invitrogen, USA). Total RNA was reversed to cDNA using HiScript III Reverse Transriptase (Vazyme, Nanjing, China). QRT-PCR was performed on an AB7300 thermo-recycler (Applied Biosystems, USA) using ChamQ SYBR qPCR Master Mix (Vazyme, Nanjing, China) to amplify cDNA with specific primers (Table 1), GAPDH and β-actin were used as an internal standard control, respectively.

**Table 1:** Sequences of the primers utilized for real-time quantitative PCR

Name	Sequence
CRYAB	
Forward (5′–3′)	CCTGAGTCCCTTCTACCTTCG
Reverse (5′–3′)	CACATCTCCCAACACCTTAACTT
TFRC	
Forward (5′–3′)	AGGTGTTGGGAGATGTGATTGA
Reverse (5′–3′)	GGATGAAGTAATGGTGAGAGGGT
SQLE	
Forward (5′–3′)	GGCATTGCCACTTTCACCTAT
Reverse (5′–3′)	GGCCTGAGAGAATATCCGAGAAG
G6PD	
Forward (5′–3′)	CGAGGCCGTCACCAAGAAC
Reverse (5′–3′)	GTAGTGGTCGATGCGGTAGA
GAPDH	
Forward (5′–3′)	TGTGGGCATCAATGGATTTGG
Reverse (5′–3′)	ACACCATGTATTCCGGGTCAAT
β-actin	
Forward (5′–3′)	CATGTACGTTGCTATCCAGGC
Reverse (5′–3′)	CTCCTTAATGTCACGCACGAT

### Western blot analysis

Cells were lysed with radio immunoprecipitation assay buffer (RIPA, Beyotime, China) supplemented with PMSF (Sigma), and the protein concentration was determined using the bicinchoninic acid (BCA) protein assay (Beyotime, China). Protein samples (20 µg) were mixed with Bolt LDS sample buffer (Invitrogen) and incubated for 10 min at 95 °C. Samples were run on TGX FastCast acrylamide gels (Bio-Rad, USA) and electrophoretically transferred to PVDF membranes (Millipore, USA). Membranes were incubated at 4 °C overnight with primary antibody, and then with secondary antibody for 2 h at room temperature. Primary antibodies used in the study were rabbit anti-TFRC (1:1000, ProteinTech, Wuhan, China) and mouse anti-GAPDH (1:1000, Cell Signaling). Finally, the blots were then incubated with chemiluminescence substrate, and then visualized using Bio-Rad chemiluminescence imaging system.

### Cell proliferation assay

For cell proliferation assay, a total of 1 × 103 transfected cells were planted into each well of 96-well plates. The cell viability was assessed at 0, 24, 48, 72, and 96 h after seeding by the cell counting kit-8 (CCK-8) system (Dojindo, Japan) according to the manufacturer’s instructions. The optional density (OD) of each well was measured at 450 nm (Tecan, Switzerland).

### Cell colony formation assay

For colony formation assay, a total of 400 transfected cells were planted into each well of 6-well plates and cultured in a DMEM medium containing 10% percent FBS for about 2 weeks until colony was obviously formed. Then, the cells were fixed with methanol for 20 min, stained with 0.1% percent crystal violet for 30 min and counted.

### Apoptosis assay

Cell apoptosis was assessed by using Annexin V-FITC Apoptosis Detection Kit (Beyotime, Shanghai, China) according to the manufacturer’s instructions. Flow cytometry was performed using a Gallios flow cytometer (Beckmann Coulter, USA) and data were analyzed using Kaluza software (Beckman Coulter, USA).

### Statistical analysis

In our study, statistical analyses were performed by utilizing R software (Version 4.0.3) and IBM SPSS Statistics (Version 26.0) and. The K‐M survival analysis of two risk groups was carried out by utilizing the log-rank test. PCA and t-SNE analyses were performed using the “stats” and “Rtsne” R package, respectively. The correction between the FAGs signature (riskScore) and immune cells was analyzed by Spearman correlation analysis. All statistical tests were two-sided and P < 0.05 was considered statistically significant.

## Results

### Identification of differently expressed FAGs (DEFAGs)

**Fig. 1:**
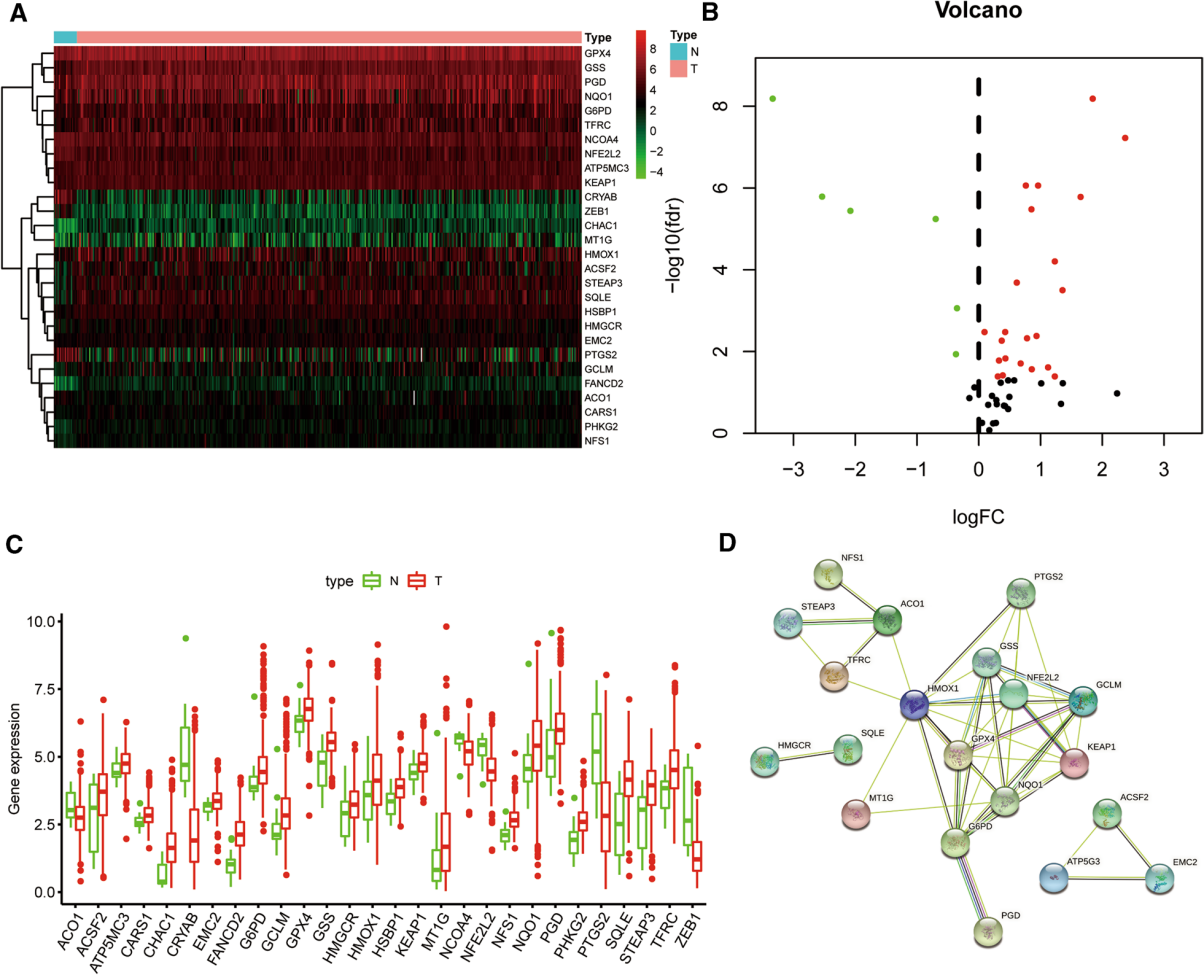
Differently expressed ferroptosis-associated genes (DEFAGs) in Bladder cancer (BLCA). **A** Heatmap of DEFAGs; **B** Volcano map of DEFAGs; **C** Boxplot of DEFAGs; **D** PPI network of DEFAGs

The RNA-sequencing data of BLCA patients and corresponding clinicopathological information were acquired from TCGA (Additional file 12: Table S2). 414 BLCA and 19 normal tissue samples were finally included in our research. By setting the joint satisfaction of FDR < 0.05 as the cut-off criterion, 28 DEFAGs were screened out from a list of 60 FAGs. The heatmap of DEFAGs between normal bladder and BLCA tissues was detailed in Fig. 1A. The volcano plot presenting 22 up-regulated and six down-regulated FAGs was shown in Fig. 1B. The Boxplot of DEFAGs was demonstrated in Fig. 1C. Besides, one protein–protein interaction (PPI) network was established providing interactive information among these DEFAGs (Fig. 1D).

We also performed network analysis of TFs-FAGs interaction. The heatmap of DETFs was demonstrated in Additional file 1: Figure S1A, B was a volcano map presenting 40 up-regulated and 36 down-regulated DETFs. Moreover, the DETFs-DEFAGs interaction network was displayed in Additional file 1: Figure S1C.

### Functional annotation and pathway enrichment of differently expressed FAGs (DEFAGs)

**Fig. 2:**
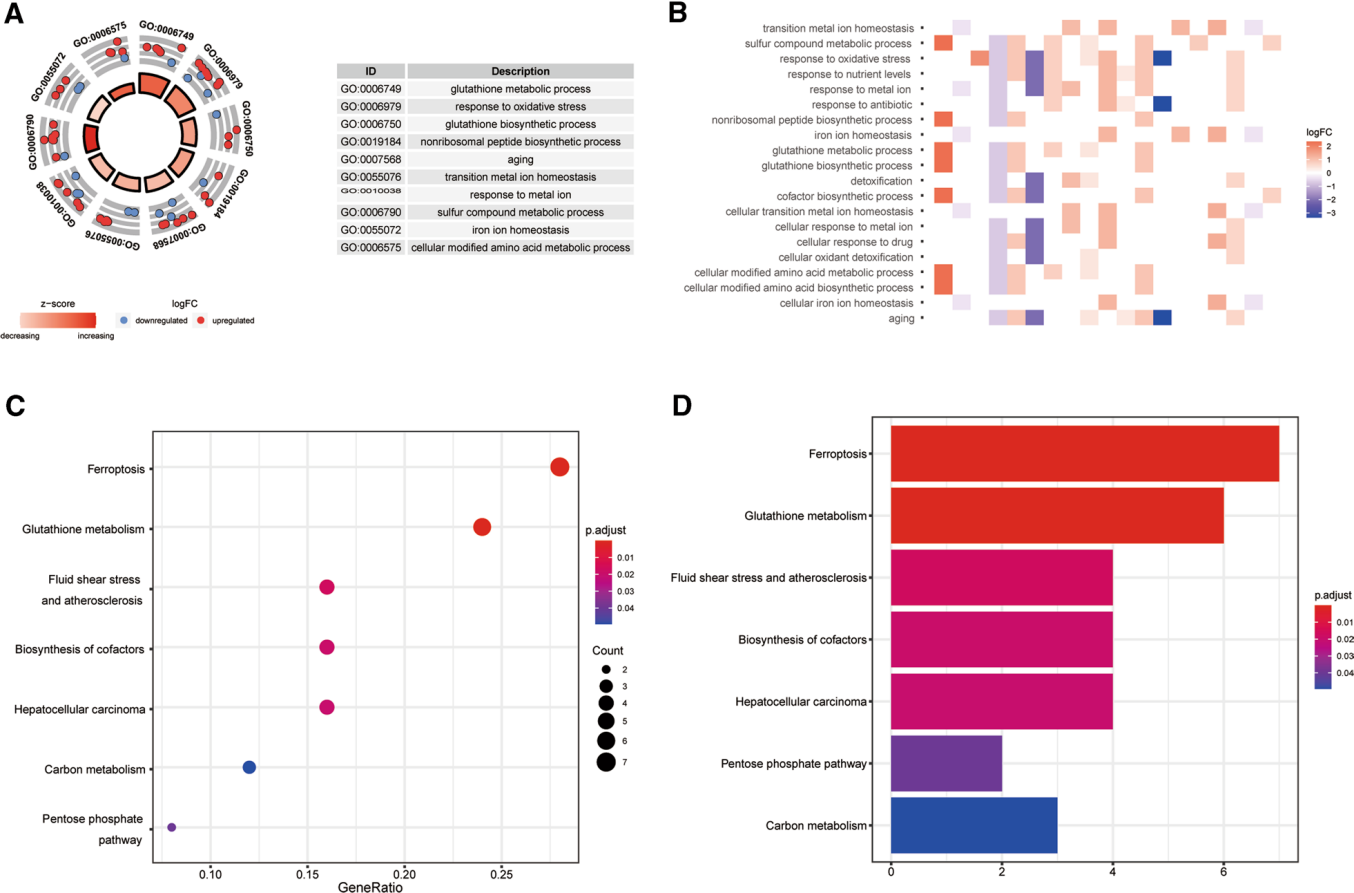
Functional annotation of differently expressed ferroptosis-associated genes (DEFAGs). **A** Circle diagram of Gene ontology (GO) enrichment analysis. Blue circles indicate down-regulation, whereas red ones indicate up-regulation; **B** Heatmap of GO enrichment analysis; **C**, **D** The bubble plot (**C**) and bar plot (**D**) of KEGG pathways

To better comprehend the potential molecular mechanisms and functions of DEFAGs, GO and KEGG terms analysis were carried out. GO enrichment analysis demonstrated that DEFAGs were mainly enriched in the biological processes (BP) associated with response to oxidative stress, aging, response to metal ion, sulfur compound metabolic process, glutathione metabolic process, transition metal ion homeostasis, cellular modified amino acid metabolic process, iron ion homeostasis, glutathione biosynthetic process and non-ribosomal peptide biosynthetic process. Concerning the molecular function (MF), we found that the DEFAGs were mainly concentrated in coenzyme binding, ligase activity, NADP binding, antioxidant activity, oxidoreductase activity, acting on CH–OH group of donors oxidoreductase activity, acting on the CH–OH group of donors, NAD or NADP as acceptor, oxidoreductase activity, acting on paired donors, with incorporation or reduction of molecular oxygen, acid—amino acid ligase activity, ligase activity, forming carbon—nitrogen bonds and peroxidase activity (Fig. 2A, B, Additional file 13: Table S3). Moreover, in terms of KEGG pathway analysis, the results showed that the DEFAGs were significantly enriched in ferroptosis, glutathione metabolism, fluid shear stress and atherosclerosis, Biosynthesis of cofactors, hepatocellular carcinoma, carbon metabolism and pentose phosphate pathway. (Fig. 2C, D, Additional file 14: Table S4).

### Construction of four FAGs established signature (riskScore)

**Fig. 3:**
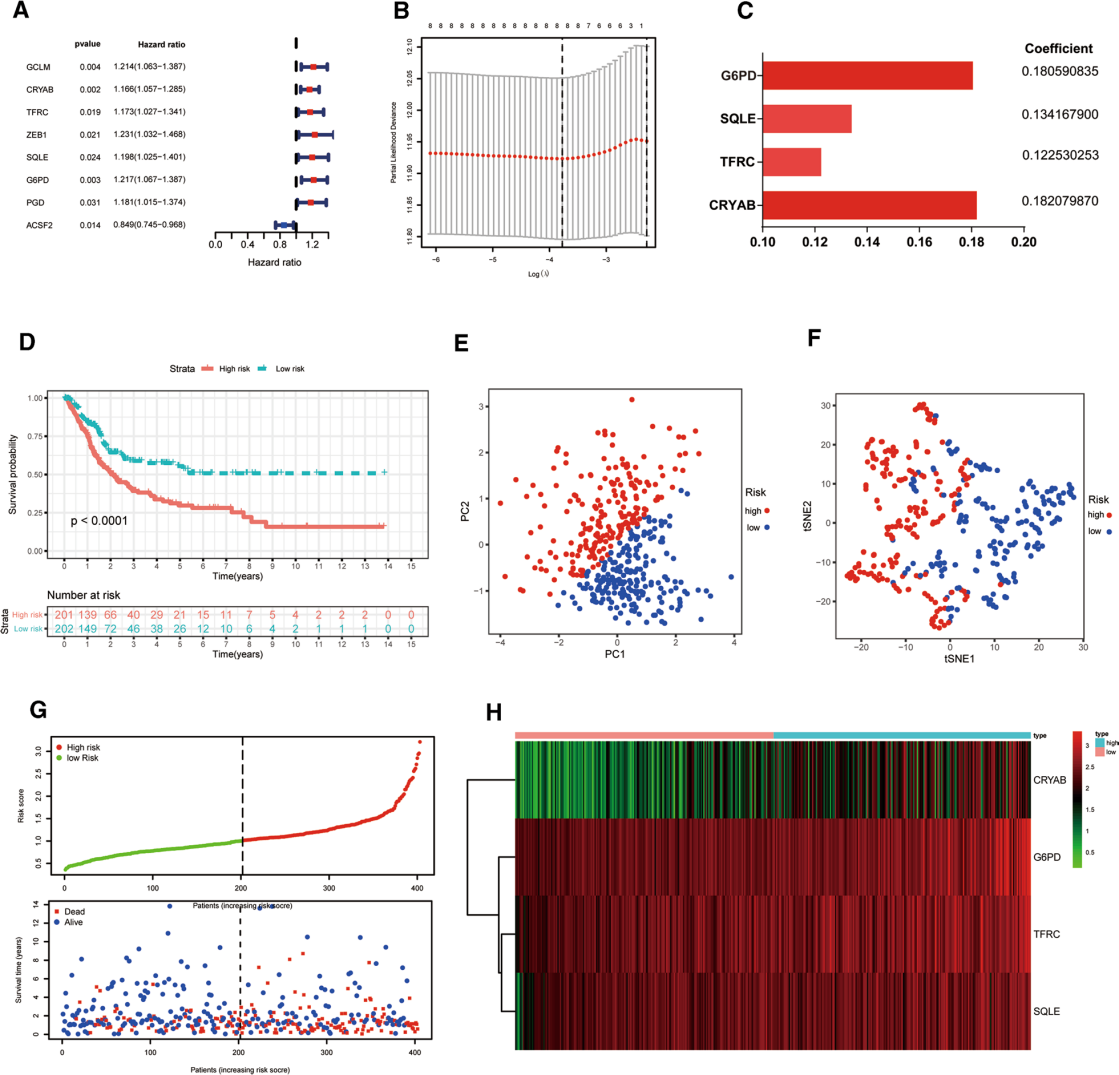
Construction of the ferroptosis-associated genes (FAGs) signature in accordance with the TCGA cohort. **A** The forest plot of the univariate Cox; **B** The cross-validation fit plot of LASSO Cox analysis; **C **The coefficients of four vital FAGs measured by the multivariate Cox; **D** Kaplan–Meier plot; **E** PCA plot; **F** t-SNE analysis; **G** The riskScore distribution and survival status distribution of BLCA patients in two-risk groups; **H** Heatmap of four crucial FAGs

The univariate Cox regression analysis was applied to assess the prognostic value of DEFAGs, which then displayed that eight FAGs were significantly linked to OS in BLCA patients (P < 0.05) (Fig. 3A, Additional file 15: Table S5). Next, the LASSO regression analysis was used to avoid overfitting (Fig. 3B). To further explore FAGs with the greatest prognosis value, the multivariate Cox regression analysis was utilized, and four hub FAGs (CRYAB, TFRC, SQLE and G6PD) were eventually selected to constructed the FAGs signature (riskScore) (Fig. 3C, Additional file 16: Table S6). The four prognostic FAGs related formula was measured as follows: riskScore = (0.182079870 × ExpCRYAB) + (0.122530253 × ExpTFRC) + (0.134167900 × ExpSQLE) + (0.180590835 × ExpG6PD). In addition, the genetic alteration status was investigated according to the cBioPortal database. The results indicated that CRYAB, TFRC, SQLE and G6PD had 0.5%, 6%, 6% and 3% genetic alterations, respectively (Additional file 2: Figure S2). In accordance with the formula described above, the riskScore of each BLCA patient in the TCGA database was calculated. Next, based on the median value of our riskScore, we distributed BLCA patients into two risk groups (low-risk and high-risk). Then, K-M survival curve was plotted to estimate the power of model in predicting patients’ clinical results, and the results uncovered that BLCA patients with low-risk had a better OS than those with the high-risk (P = 2.787e−05, Fig. 3D). PCA plot and t-SNE analysis displayed that samples in two risk groups were distributed in two directions (Fig. 3E, F). Besides, we found that with the increase of riskScore, the number of patients in the high-risk group grew, and dead events increased (Fig. 3G). A heatmap of four hub FAGs expression was demonstrated in Fig. 3H. Moreover, to better assess the prognostic value of our four FAGs established signature (riskScore), the time-dependent ROC analysis was performed, and AUC reached 0.629 at 3-year and 0.653 at 5-year, which showed the moderate prediction accuracy (Additional file 3: Figure S3A, B).

### Independent prognostic and clinicopathological correlation analyses

**Fig. 4:**
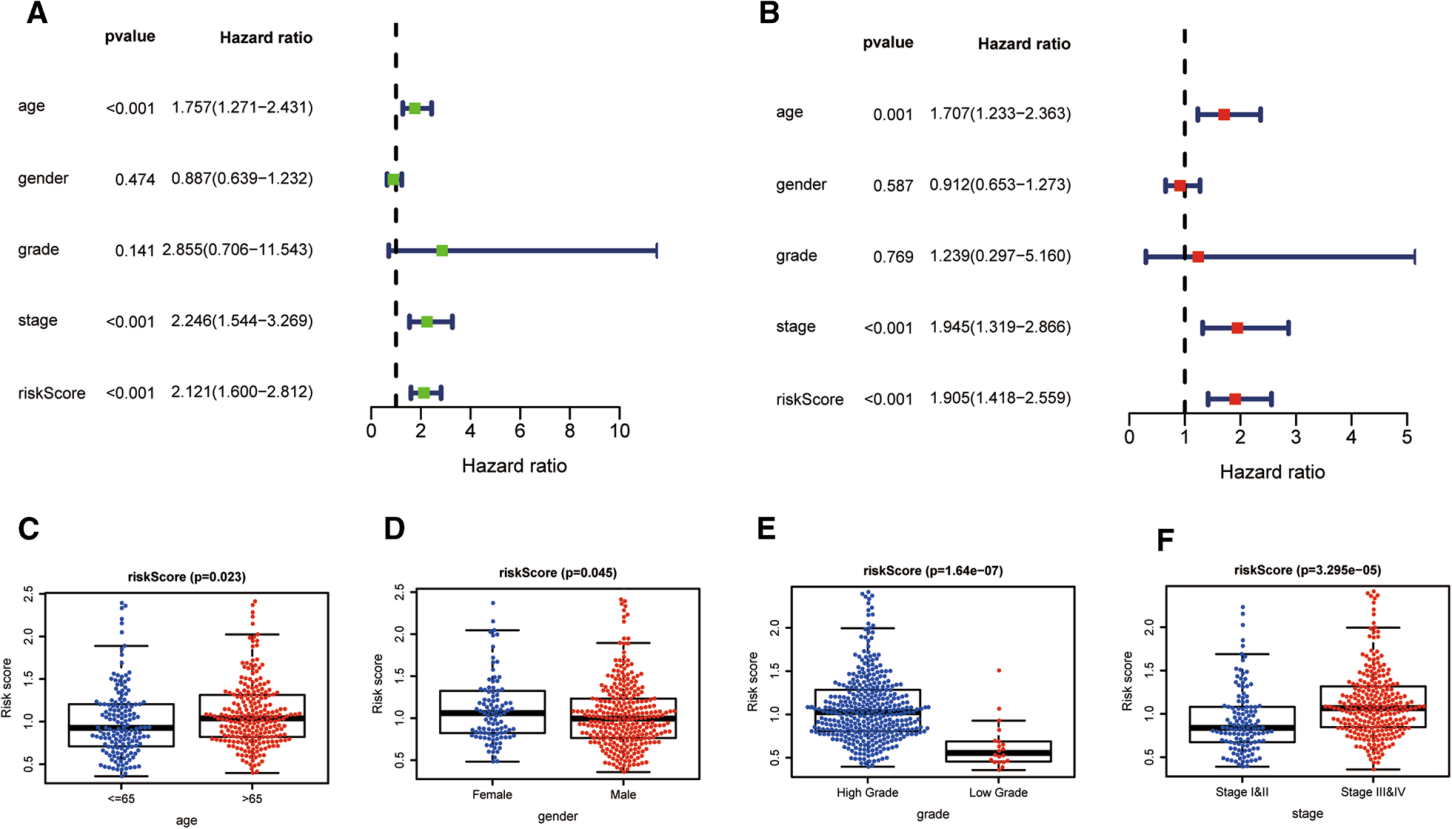
The relationship between the riskScore and clinical factors; **A** The univariate Cox analysis demonstrated that age, stage and riskScore were statistically different; **B** The multivariate Cox analysis displayed that riskScore was an independent prognostic parameter; **C**–**F** The scatter diagrams showed that age (**C**), gender (**D**), grade (**E**), and stage (**F**), were strongly related to the riskScore

**Fig. 5:**
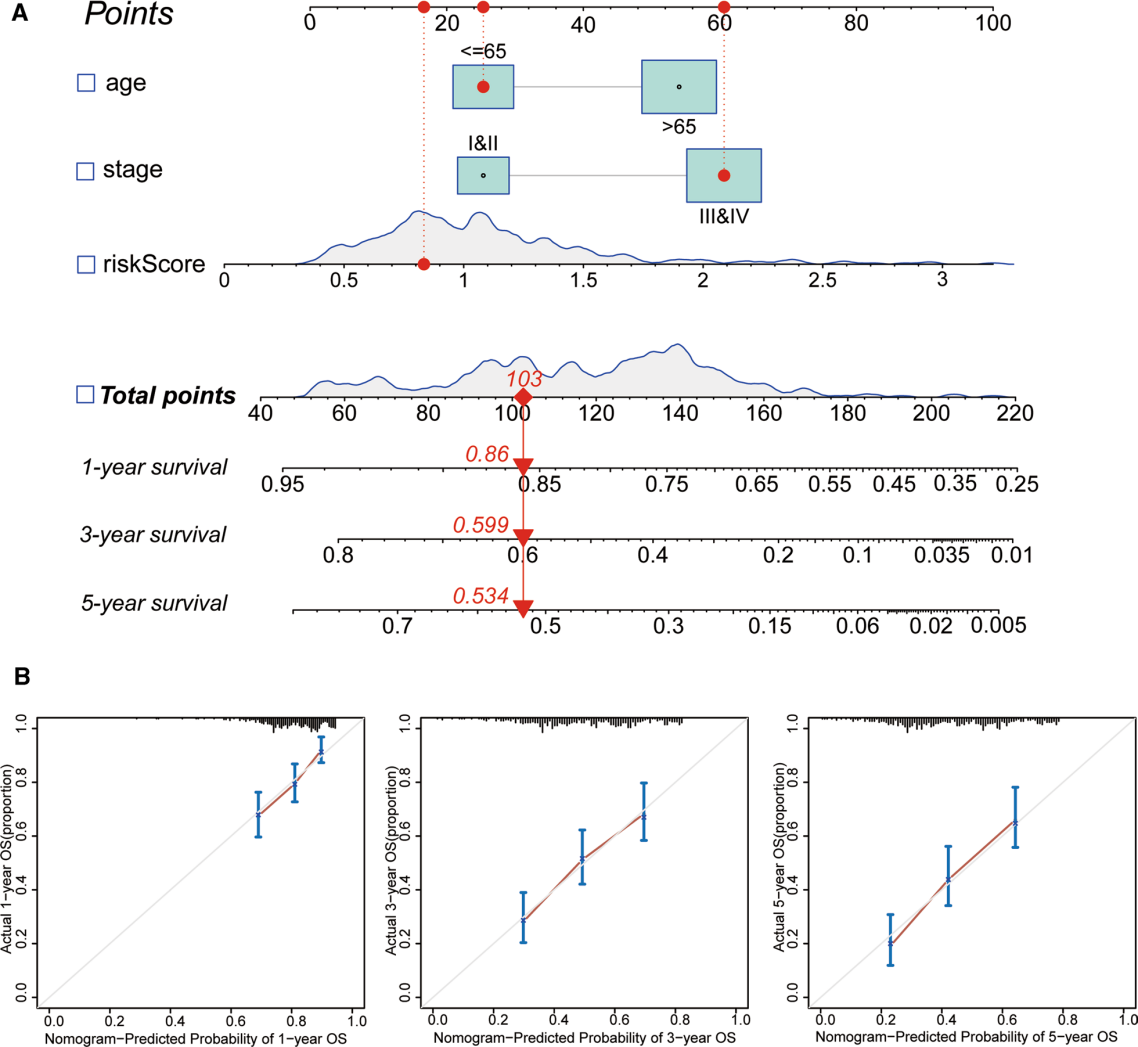
Construction of a nomogram based on the ferroptosis-associated genes (FAGs) signature. **A** The nomogram combined with the variables (age, stage and riskScore). **B** Calibration curve of the nomogram at 1, 3, and 5 years

To determine whether the FAGs based signature could serve as an independent variable in BLCA, the riskScore and several clinicopathologic parameters were included. As shown in Fig. 4A and B, our established model (riskScore) remained significant through the univariate and multivariate Cox regression analyses (both P < 0.001). Simultaneously, we also explored the relationship between clinicopathologic characteristics and riskScore. The results uncovered that the FAGs based signature was firmly associated with age (P = 0.023), gender (P = 0.045), grade (P = 1.64e−07) and stage (P = 3.295e−05) (Fig. 4C–F). Moreover, a prognostic nomogram incorporating clinical parameters (age and stage) and the FAGs based signature was built, which could predict BLCA patients’ survival probability (Fig. 5A). The calibration curve also demonstrated the excellent reliability and veracity of the hybrid nomogram (Fig. 5B).

### The correlation between the FAGs based signature (riskScore) and immunity

**Fig. 6:**
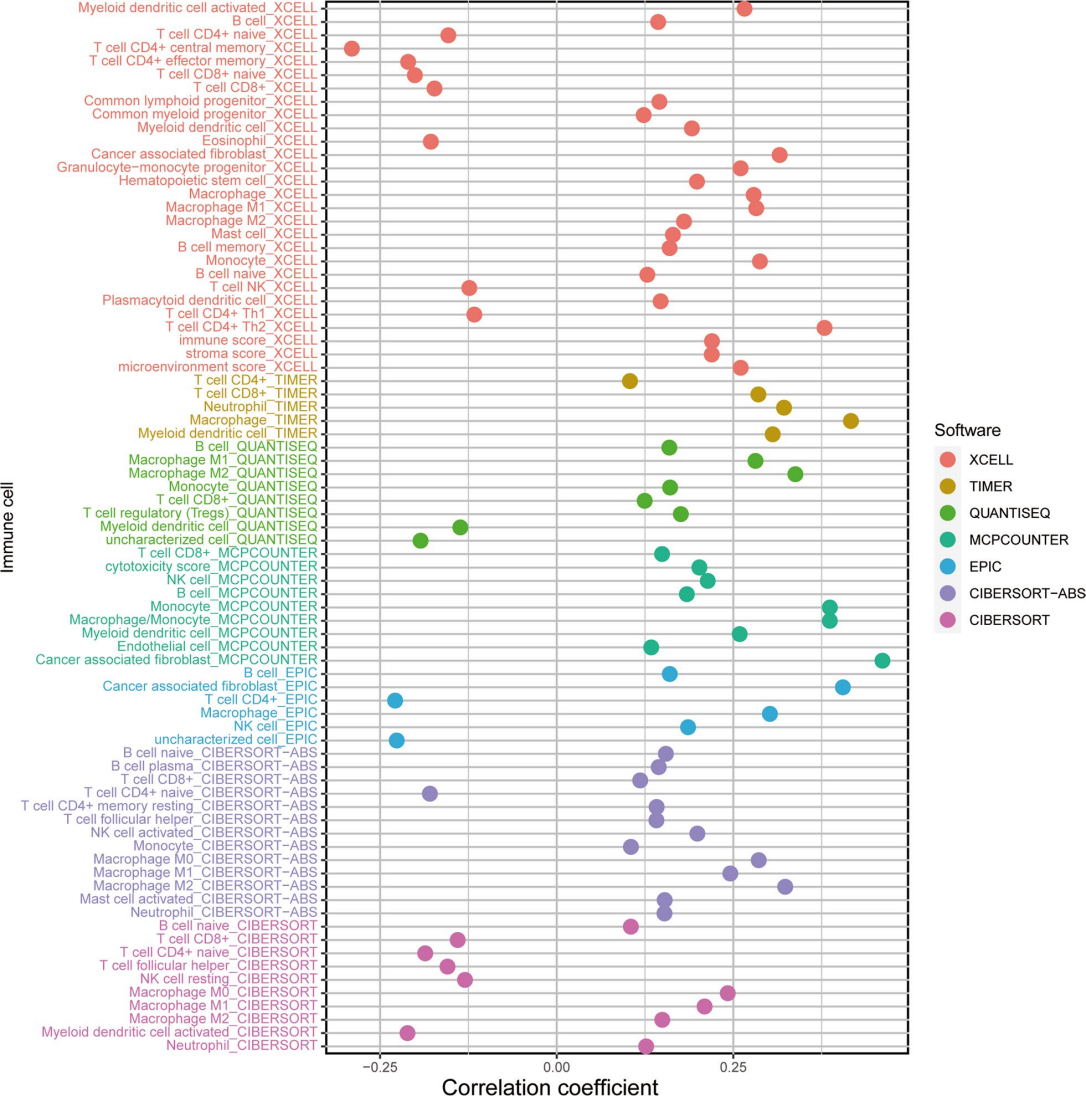
Estimation of tumor-infiltrating immune cells by the ferroptosis-associated genes (FAGs) signature based on XCELL, TIMER, QUANTISEQ, MCPCOUNT, EPIC, CIBERSORT and CIBERSORT-ABS algorithms

**Fig. 7:**
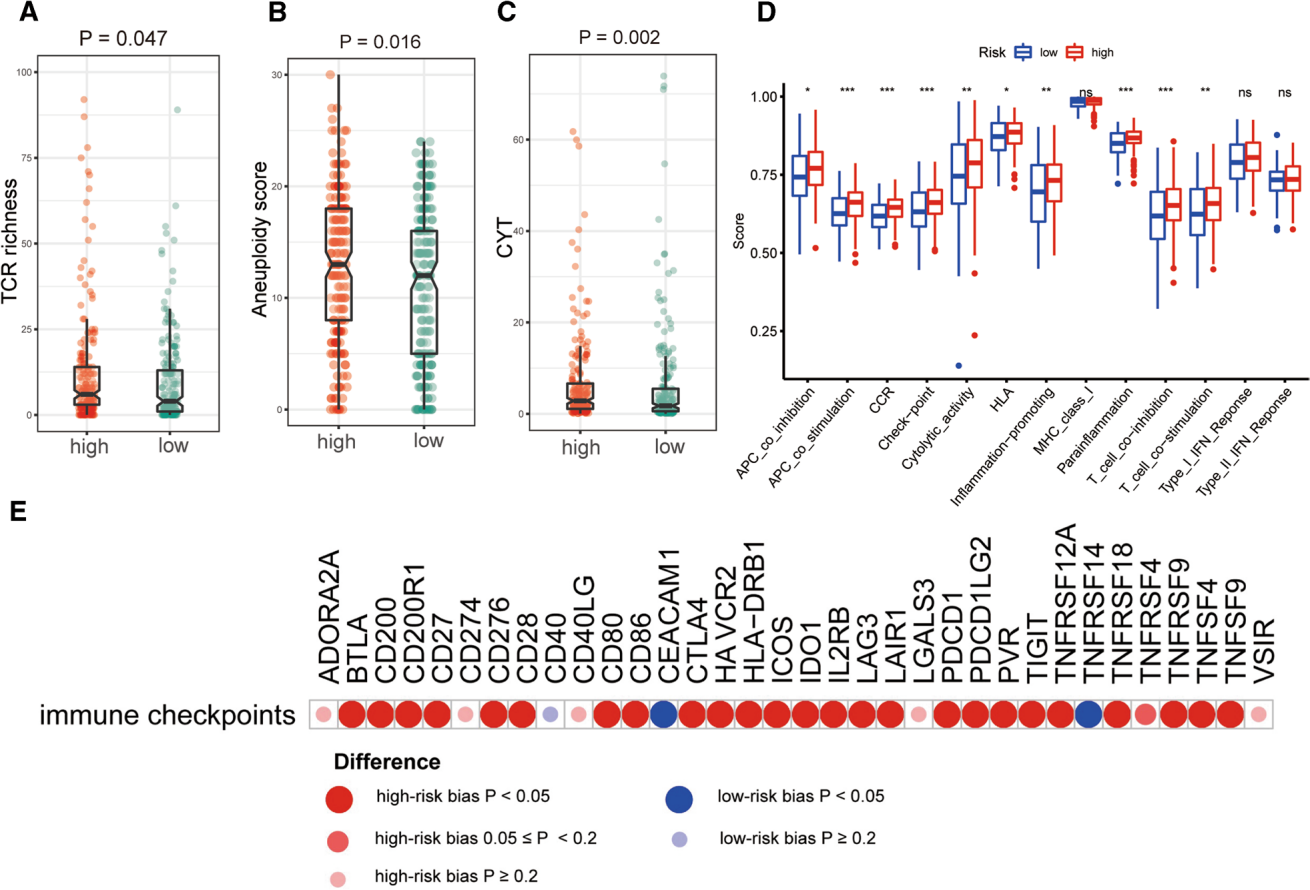
Estimation of immune-related biomarkers by the ferroptosis-associated genes (FAGs) signature. **A**–**C** Differences in TCR richness (**A**), CYT (**B**), and Aneuploidy score (**C**) between two risk groups (high-risk and low-risk); **D** Differences in ssGSEA scores of 13 immune-related functions; *P < 0.05; **P < 0.01; ***P < 0.001; E Differences in mRNA expression of thirty-four immune checkpoints

In our study, the immune infiltration scores were calculated by utilizing XCELL, TIMER, QUANTISEQ, MCPCOUNT, EPIC CIBERSORT and CIBERSORT-ABS algorithms. Patients in the high-risk group were positively related to monocyte and macrophage, but they were negatively connected with myeloid dendritic cell activated, CD4 + T cell and so on (Additional file 4: Figure S4, Additional file 5: Figure S5). A specific Spearman correlation analysis was performed, and the results was shown in Fig. 6 and Additional file 17: Table S7. Next, we included several significant biomarkers utilized in patients with immunotherapy, such as CYT, TCR, aneuploidy score and thirty-four immune checkpoints. As shown in Fig. 7A, TCR richness scores were higher in the BLCA high-risk group (P < 0.047). Regarding ICB treatment-related biomarkers, the results indicated that aneuploidy score (P = 0.016) and CYT (P = 0.002) were higher in the BLCA high-risk group, too (Fig. 7B, C). With respect to the differences of thirty-four immune checkpoints-related biomarkers, we found that a majority of immune checkpoints, such as CD200R1, HAVCR2, PDCD1LG2 and TNFSF9 also demonstrated the high-risk group biased (Fig. 7E).

Further, by utilizing the ssGSEA method, enrichment scores of the immune related pathways were quantified. Interestingly, most of antigen presentation-related functions, such as APC co-inhibition, HLA, and T cell co-stimulation displayed the high-risk group biased (Fig. 7D). Moreover, we also found that differently expressed genes between two risk groups were also significantly enriched in some immune-related biological processes or pathways, including cytokine-cytokine receptor interaction pathway, immunoglobulin receptor binding and so on (Additional file 6: Figure S6, Additional file 7: Figure S7).

### Verification of the prognostic model based on the GEO cohort

**Fig. 8:**
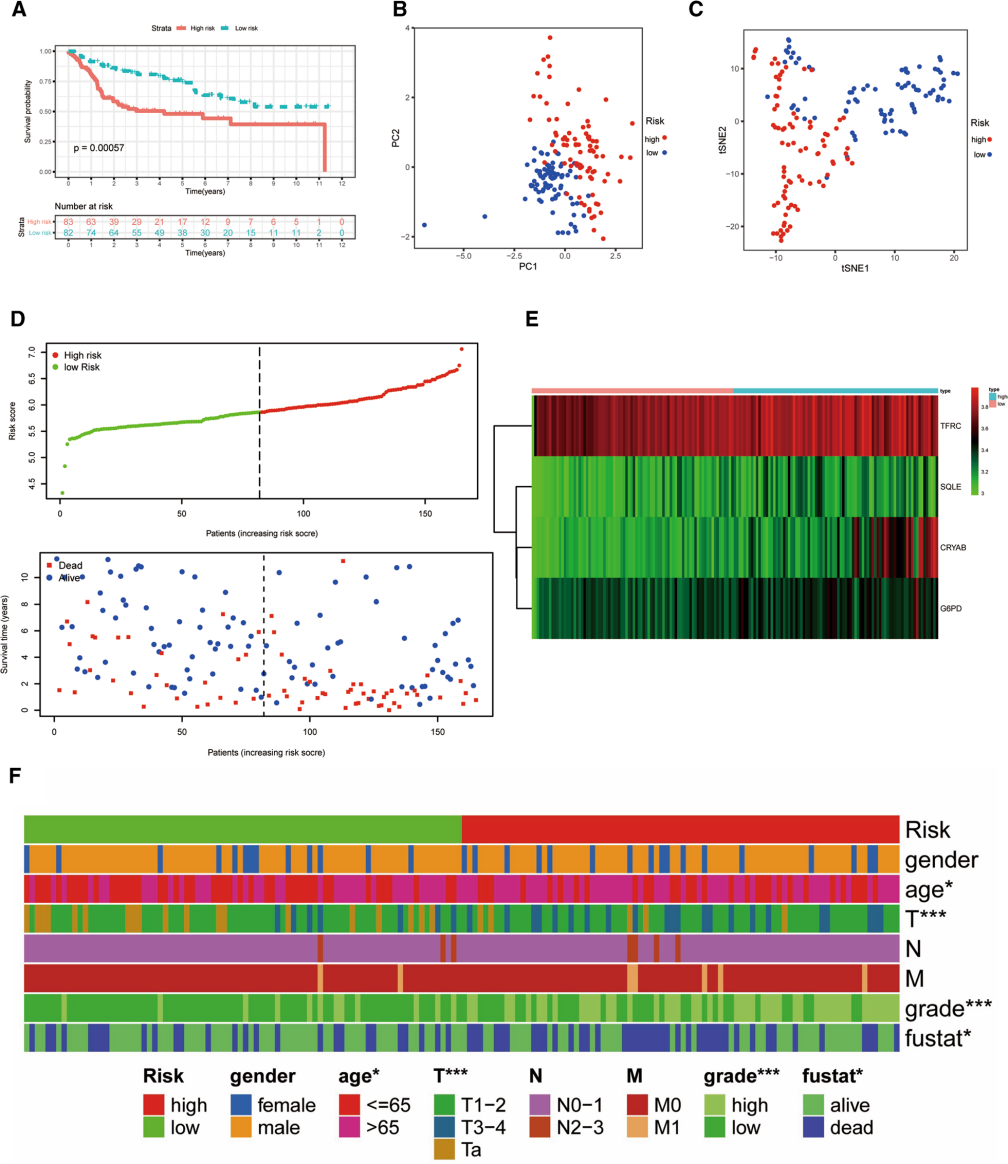
Verification of the four ferroptosis-associated genes (FAGs) signature in the GSE13507 cohort. **A** Kaplan–Meier plot; **B** PCA plot; **C** t-SNE analysis; **D** The riskScore distribution and OS status distribution; **E** The heatmap of four crucial FAGs; **F** A strip chart indicated that age, T stage and tumor grade were significantly related to riskScore. *P < 0.05; ***P < 0.001

To validate the stability and reliability of the FAGs signature, we downloaded 165 BLCA patients with clinicopathological parameters as the validation database from the GSE13507 cohort (Additional file 18: Table S8). Samples were also categorized into two risk groups on the basis of the same formula from TCGA, and K-M survival analysis uncovered that the low-risk group has a poor survival rate (P = 8.252e−04) (Fig. 8A). Consistent with the outcomes in TCGA, t-SNE and PCA analyses suggested that BLCA samples were distributed into a discrete direction in two risk groups (Fig. 8B, C). Likewise, with the increase of riskScore, the number of high-risk patients grew, and dead events increased (Fig. 8D). The heatmap of 4 critical FAGs in the GSE13507 database was also revealed in Fig. 8E. The ROC analysis indicated that our riskScore had the predictive power for the prognosis in BLCA (Additional file 3: Figure S3C, D). Next, a heatmap was created to reveal the potential relevance of the FAGs signature and clinicopathological factors (Fig. 8F). The results displayed that age, T stage and grade were strongly relevant to the riskScore (all P < 0.05) (Additional file 8: Figure S8).

### Verification of the crucial FAGs in the signature

**Fig. 9:**
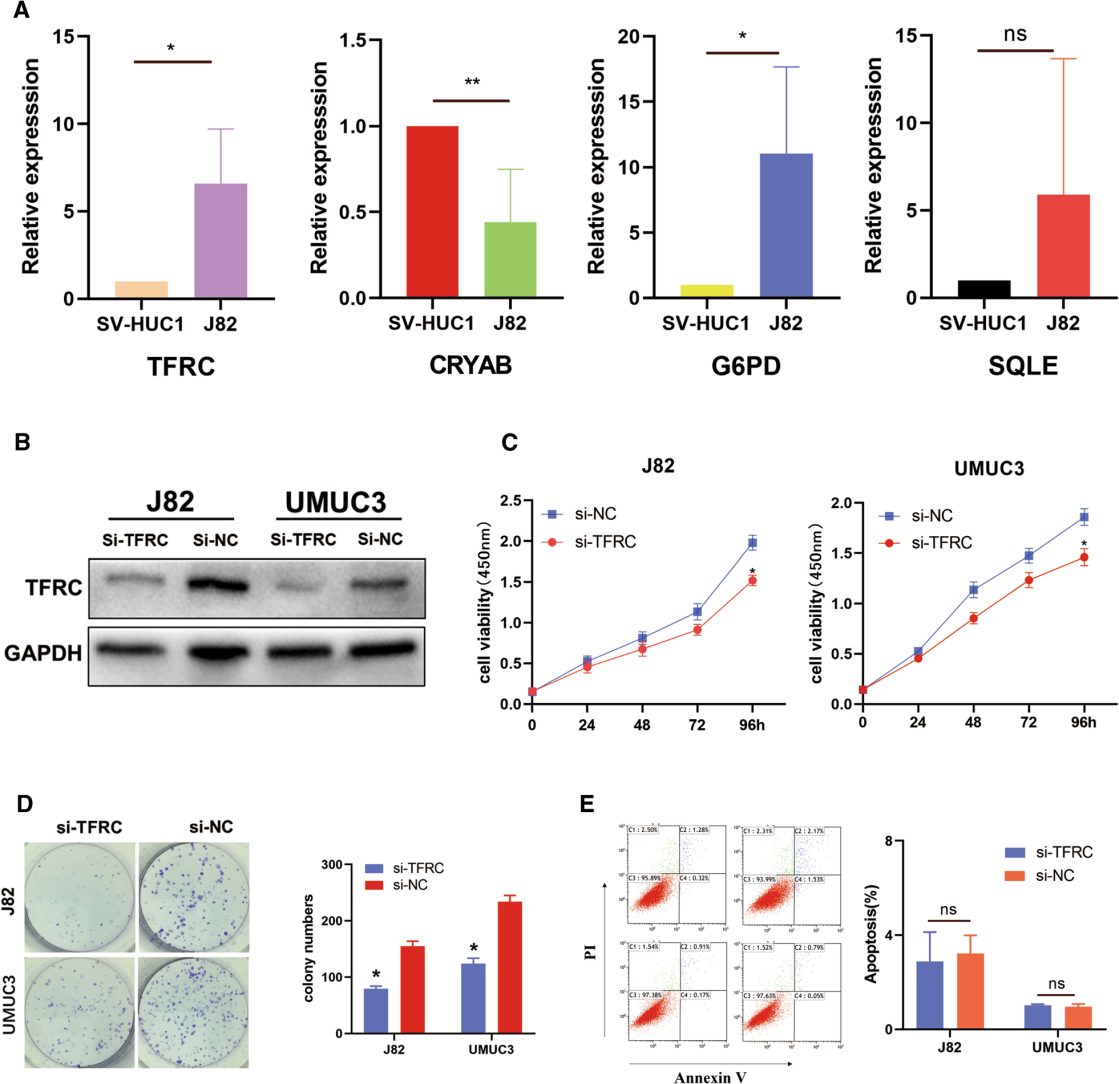
Verification of the crucial ferroptosis-associated genes (FAGs) in the signature. **A** The mRNA levels of four FAGs (TFRC, SQLE, G6PD and CRYAB) in J82 and SV-HUC1 were detected by QRT-PCR. **B** The efficiency of TFRC siRNA was validated utilizing western blots. **C**, **D** The CCK-8 and colony formation assays indicated that down-regulation of TFRC inhibited cell proliferation in J82 and UMUC3. **E** TFRC had no effect on the apoptosis of BLCA cells. **P < 0.01; *P < 0.05

Expression levels of four crucial FAGs between tumor and normal tissue were demonstrated in Additional file 9: Figure S9, which indicated that G6PD, TFRC and SQLE were significantly upregulated in BLCA, while CRYAB was significantly downregulated when compared with normal tissues (all P < 0.05) (Additional file 9: Figure S9A) and paired non-tumor tissues (all P < 0.05) (Additional file 9: Figure S9B). We also used real-time PCR to detect mRNA expression levels of CRYAB, TFRC, SQLE, and G6PD in J82 and SV-HUC1 cell lines. Firstly, GAPDH was utilized as the internal control, and the results showed that compared to the SV-HUC1 cells, there was a down-regulation of CRYAB in the J82 cells, while the other two gene expression levels (TFRC and G6PD) upregulation. Regarding SQLE, although there was no statistically significant difference, the mRNA expression level of SQLE in the SV-HUC1 cells was higher than that in the J82 cells (Fig. 9A). Then, β-actin was utilized as the internal control, and we found that the expression levels of TFRC, SQLE and G6PD were consistent with the results when GAPDH was used as internal control (Additional file 10: Figure S10). In all, the results were basically consistent with our bioinformatics analysis (Additional Files 19, 20, 21, 22).

Considering the signature was strongly related to the OS in BLCA, the crucial FAGs might be of great impact in the biological functions. Moreover, it was reported that transferrin receptors (TFRC) played the important role in varieties of tumors, such as epithelial ovarian cancer, breast cancer, and liver cancer [21, 40, 41]. Therefore, we focused on the hub gene (TFRC) for further validation. We knocked down TFRC in J82 and UMUC3 cells by siRNA, and the efficiency was confirmed by western blots (Fig. 9B), and then evaluated the effects of TFRC on BLCA cells by using CCK-8, colony formation and apoptosis analysis. As expected, knockdown of TFRC could inhibit BLCA cell proliferation and colony formation (Fig. 9C, D). However, apoptosis assay indicated that TFRC had no effect on cell apoptosis (Fig. 9E).

### Small molecule drugs screening

To identify the candidate molecule drugs connected with FAGs in BLCA, we performed cMap database analysis. According to DEFAGs between the normal tissue and BLCA, the candidate molecule drugs were identified with strongly significant associations (|mean| > 0.5, n ≥ 4 and P < 0.001). The results indicated that seven small molecule drugs were negatively relevant to BLCA including Cycloheximide, cephaeline, puromycin, lanatoside C, anisomycin, atropine and 8-azaguanine, displaying the potential to repress BLCA. Two small molecule drugs were positively correlated with BLCA including khellin and amantadine, which indicated the potential to promote cancer development (Table 2).

**Table 2:** The results of Connectivity map (CMap) analysis

Rank	Cmap name	Mean	n	Enrichment	p	Specificity	Percent non-null
1	Cicloheximide	− 0.756	4	− 0.978	0	0	100
2	Cephaeline	− 0.616	5	− 0.916	0	0.0181	100
3	Puromycin	− 0.674	4	− 0.923	0.00004	0.0299	100
4	Khellin	0.627	5	0.881	0.00006	0	100
5	Lanatoside C	− 0.558	6	− 0.829	0.00006	0.0213	100
6	Anisomycin	− 0.632	4	− 0.907	0.0001	0.0424	100
7	Atropine	− 0.61	4	− 0.906	0.00014	0	100
8	Amantadine	0.55	4	0.878	0.0003	0	100
9	8-Azaguanine	− 0.613	4	− 0.888	0.00034	0	100

## Discussion

Since ferroptosis was reported in 2012, it has attracted much attention due to its relationship with development, senescence, immunity, and pathological processes [14]. Although ferroptosis played the pivotal role in sustaining survival of normal cells and tissues, an increasing number of studies uncovered that several oncogenic pathways had something to do with ferroptosis, making cancer cells highly vulnerable to ferroptotic death [17, 22].

In our study, the expression of sixty FAGs in BLCA were systematically analyzed. First, in accordance with the BLCA data from the TCGA dataset, 28 DEFAGs between normal tissues and bladder cancer tissues were extracted. Then, we performed GO and KEGG analyses regarding these DEFAGs. Preliminary Functional annotation and pathway enrichment analysis revealed that these FAGs were mostly concentrated in some of the most important pathways related to tumor development, such as oxidative stress and glutathione biosynthetic process. However, the potential complex mechanisms of ferroptosis in tumors required to be further explored.

Under the univariate, LASSO and multivariate regression analysis, four hub FAGs (CRYAB, TFRC, SQLE and G6PD) were eventually screened out. Further, a novel prognostic signature integrating four FAGs was constructed and verified in an external cohort (GSE13507). With regard to these four FAGs, varieties of studies demonstrated that they might serve as crucial roles in tumor invasion, even in BLCA. For example, TFRC, known as the important participant in intracellular iron transport, induced epithelial ovarian cancer (EOC) cell proliferation and metastasis via upregulating the expression level of AXIN2 [40]. Regarding SQLE, it was reported that through the p53 tumor suppressor pathway deactivation and β-catenin oncogenic pathway activation, the reduction of SQLE aggravated colorectal cancer (CRC) progression and metastasis, which identified SQLE as a prognostic biomarker in colorectal cancer aggressiveness [42]. G6PD promoted cancer progression in numerous tumor types [43–46]. Chen et al. discovered that via the ROS accumulation and the AKT pathway, the modulation of G6PD influenced bladder cancer, and high expression value of G6PD was a poor prognostic factor in BLCA [43]. CRYAB was also associated with various cancers, including breast cancer, prostate cancer and lung cancer, and CRYAB protein was significantly related to different kinds of signaling pathways, such as inflammation, oxidative stress and apoptosis [47–50].

The K-M survival analysis displayed that patients in the low-risk group were strongly related to poor prognosis in both the TCGA dataset and the GSE13507 dataset. Then, the FAGs signature was proved to be an independent parameter in BLCA by employing the univariate and multivariate Cox regression analyses. Furthermore, we explored the relationship between the FAGs signature and clinicopathologic factors. The results demonstrated that riskScore was significantly linked to age, gender, grade and stage in the TCGA cohort, which indicated that the FAGs signature was a protective biomarker that might be importantly related to tumorigenesis, development and metastasis. Additionally, to identify a novel quantitative tool for clinicians to predict BLCA patients’ survival probability and improve risk stratification, a prognostic nomogram was constructed that incorporated the FAGs based signature and clinical parameters (age and stage). The calibration plots of our hybrid nomogram also revealed greatly satisfactory outcomes. Taken together, our work might contribute to provide new effective tools for the diagnosis and prognosis in BLCA, and offer better individualized treatment programs for patients than before.

In recent years, ferroptosis has also been proved to be associated with cancer immunotherapy. Wang et al. found that ferroptosis was related to T cell mediated tumor immunity. In tumor cells, CD8 + T cells activated by immunotherapy strengthened ferroptosis specific lipid peroxidation [51]. Targeting tumor ferroptosis pathway was a novel therapeutic method in association with checkpoint blockade [51]. Moreover, Immunotherapy has rapidly influenced the treatment paradigm for many tumors such as lung cancer and melanoma, and preclinical data revealed that BLCA was immunogenic [52, 53]. There was an increasing body of evidence suggested that immune regulation played an important role in bladder cancer, including PD-1, PD-L1, tumor-associated macrophages, myeloid-derived suppressor cells, regulatory T cells and so on [6]. Ulrich et al. demonstrated that higher pretreatment serum PD-L1 (sPD-L1) levels were related to the poor prognosis in patients following platinum and immune checkpoint inhibitor treatments [54]. Jiang et al. revealed that M2 and PD-1-positive Tumor-associated macrophages were connected with poor clinical results in muscle invasive bladder cancer patients, and the blinding of PD-1 and CD68 could stimulate tumor growth [55]. In our study, we included significant biomarkers for immunotherapy, and the results indicated that a variety of immunotherapy-related biomarkers, including CYT, TCR, aneuploidy score and most immune checkpoints showed the high-risk group biased. It suggests that our ferroptosis-based signature is strongly associated with BLCA immunity. Besides, it is interesting that the antigen presentation processes indicated the high-risk group biased. Moreover, by utilizing different algorithms, we explored the association with the riskScore and tumor infiltrating immune cells, and patients in the high-risk group were more linked to monocyte, macrophage and so on. Therefore, it was reasonable to acknowledge that ferroptosis might have a significant association with immunity in BLCA.

By means of cMap database, seven small molecule drugs containing Cycloheximide, cephaeline, puromycin, lanatoside C, anisomycin, atropine and 8-azaguanine, were identified with potential therapeutic efficacy for BLCA patients. Two small molecule drugs (khellin and amantadine) were positively connected with BLCA, displaying the potential to promote this disease. Among these molecule drugs, many studies showed that anisomycin had an important disincentive to various of solid tumors, such as ovarian cancer, leukemia and renal carcinoma, and might be a potential chemotherapeutic drug [56]. lanatoside C suppressed cell proliferation and induced cell apoptosis in the gastric cancer cell, which were associated with ROS production and mitochondria dysfunction [57]. Nayoung et al. reported that 8-Azaguanine, known as an inhibitor of the purine nucleotide biosynthesis, greatly increased the cytotoxicity of Natural killer cells and was superior to known novel immunomodulatory drugs, such as amphotericin B and fluoxetine [58]. In all, our analysis offered the possibility of several potential molecular targets for BLCA, but the efficacy and mechanism of these drugs for treatment of BLCA remained to be illustrated in the future.

We further verified the differential expression of the four FAGs that constitute the prognostic model in tumor cells through QRT-PCR, and results were consistent with the differences in gene expression levels downloaded from public databases. Then, the high genetic alteration rate gene TFRC was chosen for further verification. TFRC played critical roles in cell iron absorption. Iron deficiency could inhibit cell growth and promote cell apoptosis [59]. In this study, we found that TFRC was overexpressed in BLCA, and was linked to the poor prognosis in BLCA patients. Considering that the TFRC mRNA level of BLCA patients was higher than that of normal tissues, we decided to knock out TFRC and assessed its effect on BLCA cells. CCK-8 experiments and clone formation showed that down-regulation of TFRC inhibited BLCA cell proliferation.

The strength of this study was that it was the first time for us to perform a systematic analysis of FAG roles in BLCA with a robust statistical approach. Nonetheless, there are several limitations in our study. Firstly, the risk model was constructed and validated both based on public databases, and our results should be verified in more prospective investigations. Secondly, we only paid attention to the effect of TFRC on biological functions in bladder cancer, and the specific mechanisms should be further explored in vivo and vitro. Nevertheless, the predictive value of the signature in BLCA patients could not be ignored.

## Conclusions

Taken together, our study identified 28 differently expressed ferroptosis-associated genes (DEFAGs) and successfully constructed an individualized BLCA signature (riskScore), which proved to be significantly associated with OS in both the derivation and validation datasets. We also estimated the potential relationship among immune cell infiltration, immunotherapy-related biomarkers and immune-related pathways. Simultaneously, small molecule drugs related to FAGs were also identified for BLCA. Last but not least, TFRC was verified to be an oncogenic factor in BLCA cell lines. Our research was anticipated to provide new insights into ferroptosis for future work.

## Supplementary Information

**Additional file 1: Figure S1.** Differently expressed transcription factors (DETFs) between BLCA tissues and no-cancer tissues; (A-B) Heatmap (A) and volcano map (B) of DETFs; (C) Network reflecting the correlations between DETFs and differently expressed ferroptosis-associated genes (DEFAGs).

**Additional file 2: Figure S2.** Genetic alterations of four crucial ferroptosis-associated genes (FAGs).

**Additional file 3: Figure S3.** Time-dependent ROC analysis in the TCGA cohort and GSE13507 cohort. (A-B) The area under the curve (AUC) at 3-year (A) and at 5-year (B) in the TCGA cohort. (C-D) AUC at 3-year (C) and at 5-year (D) in the GSE13507 cohort.

**Additional file 4: Figure S4.** The representative results of the evaluation of tumor infiltrating immune cells with the FAGs signature (riskScore) model based on XCELL and QUANTISEQ algorithms.

**Additional file 5: Figure S5.** The representative results of the evaluation of tumor infiltrating immune cells with the FAGs signature (riskScore) based on TIMER, MCPCOUNT, EPIC, CIBERSORT and CIBERSORT-ABS algorithms.

**Additional file 6: Figure S6.** GO enrichment analysis of differently expressed ferroptosis-associated genes (DEFAGs) between two risk groups.

**Additional file 7: Figure S7.** KEGG enrichment analysis of differently expressed ferroptosis-associated genes (DEFAGs) between two risk groups.

**Additional file 8: Figure S8.** The scatter diagram indicated that age (A), grade (B) and stage T (C) were significantly associated with the riskScore.

**Additional file 9: Figure S9.** The expression levels of four hub FAGs (CRYAB, TFRC, SQLE and G6PD) in the signature. (A) mRNA expression in total BLCA and normal tissues; (B) mRNA expression in BLCA and paired bladder tissues. ***, P < 0.001; **, P < 0.01.

**Additional file 10: Figure S10.** The expression levels of four hub FAGs (CRYAB, TFRC, SQLE and G6PD) were verified by setting β-actin as an internal control in the QRT-PCR experiment. ***, P < 0.001; **, P < 0.01.

**Additional file 11: Table S1**. A total of 60 ferroptosis-associated genes (FAGs).

**Additional file 12: Table S2**. Clinical characteristics of BLCA patients in the TCGA cohort.

**Additional file 13: Table S3.** GO analysis of differently expressed ferroptosis -related genes.

**Additional file 14: Table S4.** KEGG analysis of differently expressed ferroptosis-associated genes.

**Additional file 15: Table S5.** The individual HR and P value of ferroptosis-associated genes (FAGs) in the TCGA dataset according to the univariate Cox analysis.

**Additional file 16: Table S6.** The regression coefficients and HR of four ferroptosis-associated genes (FAGs) according to the multiple stepwise Cox regression analysis.

**Additional file 17: Table S7.** The detail comparison results of the correlation between tumor-infiltrating immune cells and riskScore.

**Additional file 18: Table S8.** Clinical characteristics of BLCA patients in the GSE13507 dataset.

**Additional file 19.** The raw data 1 of QRT-PCR results by setting β-actin as an internal control.

**Additional file 20.** The raw data 2 of QRT-PCR results by setting β-actin as an internal control.

**Additional File 21.** The raw data 1 of QRT-PCR results by setting GAPDH as an internal control.

**Addtional file 22.** The raw data 2 of QRT-PCR results by setting GAPDH as an internal control.

## Data Availability

The raw data supporting the conclusions of this article will be made available by the authors, without undue reservation.

## Abbreviations

FAGs: Ferroptosis-associated genes
BLCA: Bladder urothelial carcinoma
TCGA: The Cancer Genome Atlas
GEO: Gene Expression Omnibus
DEFAGs: Differently expressed FAGs
DETFs: Differently expressed transcription factors
MIBC: Muscle invasive bladder cancer
NMIBC: Non-muscle invasive bladder cancer
TURB: Transurethral resection of the bladder
OS: Overall survival
GO: Gene ontology
KEGG: Kyoto encyclopedia of genes and genomes
ssGSEA: Single-sample gene set enrichment analysis
cMap: Connectivity map
PPI: Protein–protein interaction
BP: Biological processes
MF: Molecular function
