# Supplementary figures and images for: A novel signature constructed by ferroptosis-associated genes (FAGs) for the prediction of prognosis in bladder urothelial carcinoma (BLCA) and associated with immune infiltration
Source: Cancer Cell Int. 2021 Aug 6;21:414. doi: 10.1186/s12935-021-02096-3 (PMC8349026; doi:10.1186/s12935-021-02096-3)

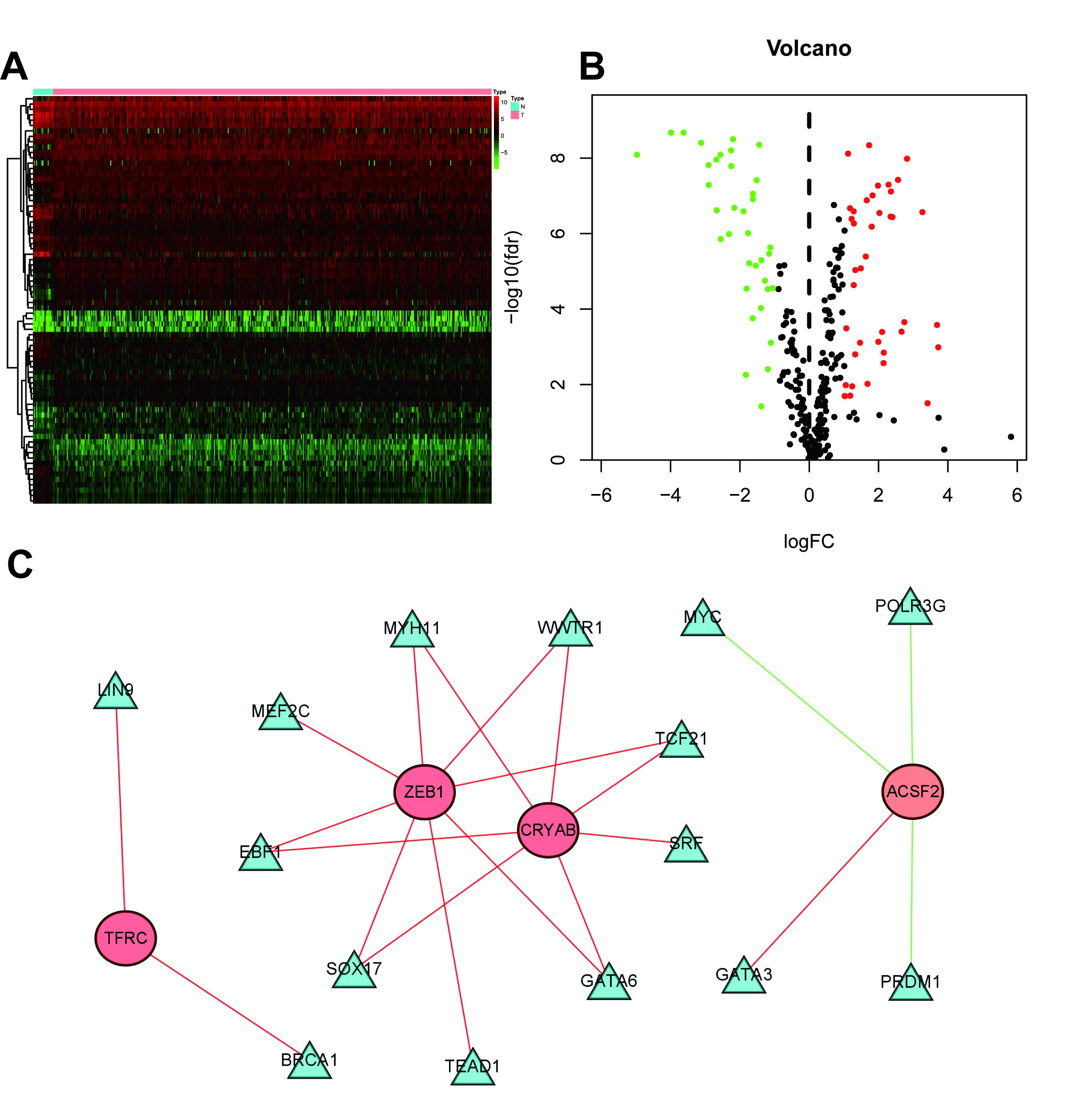

Supplement: Supplementary file 1 — Additional file 1: Figure S1. Differently expressed transcription factors (DETFs) between BLCA tissues and no-cancer tissues; (A-B) Heatmap (A) and volcano map (B) of DETFs; (C) Network reflecting the correlations between DETFs and differently expressed ferroptosis-associated genes (DEFAGs). [file 12935_2021_2096_MOESM1_ESM.tif]

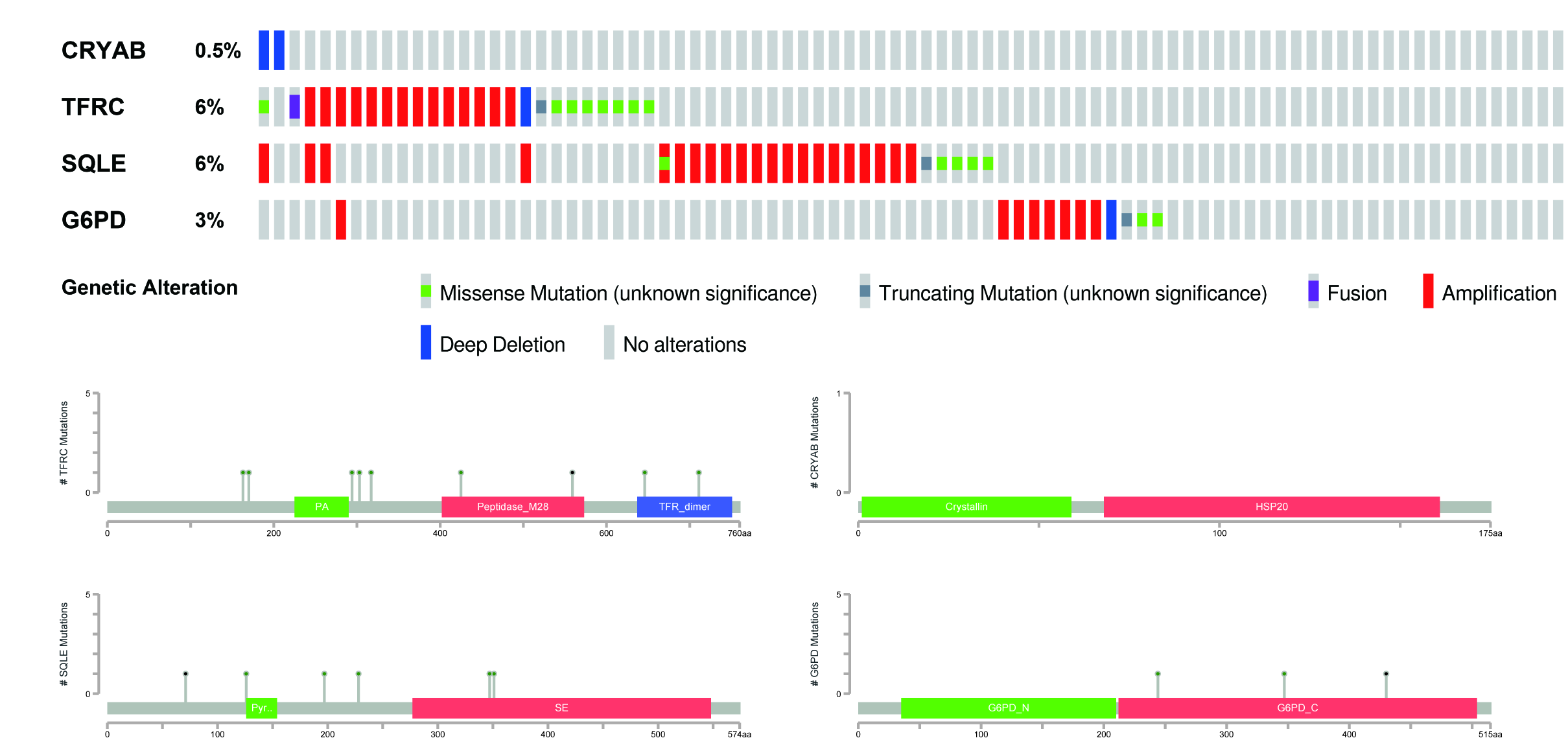

Supplement: Supplementary file 2 — Additional file 2: Figure S2. Genetic alterations of four crucial ferroptosis-associated genes (FAGs). [file 12935_2021_2096_MOESM2_ESM.tif]

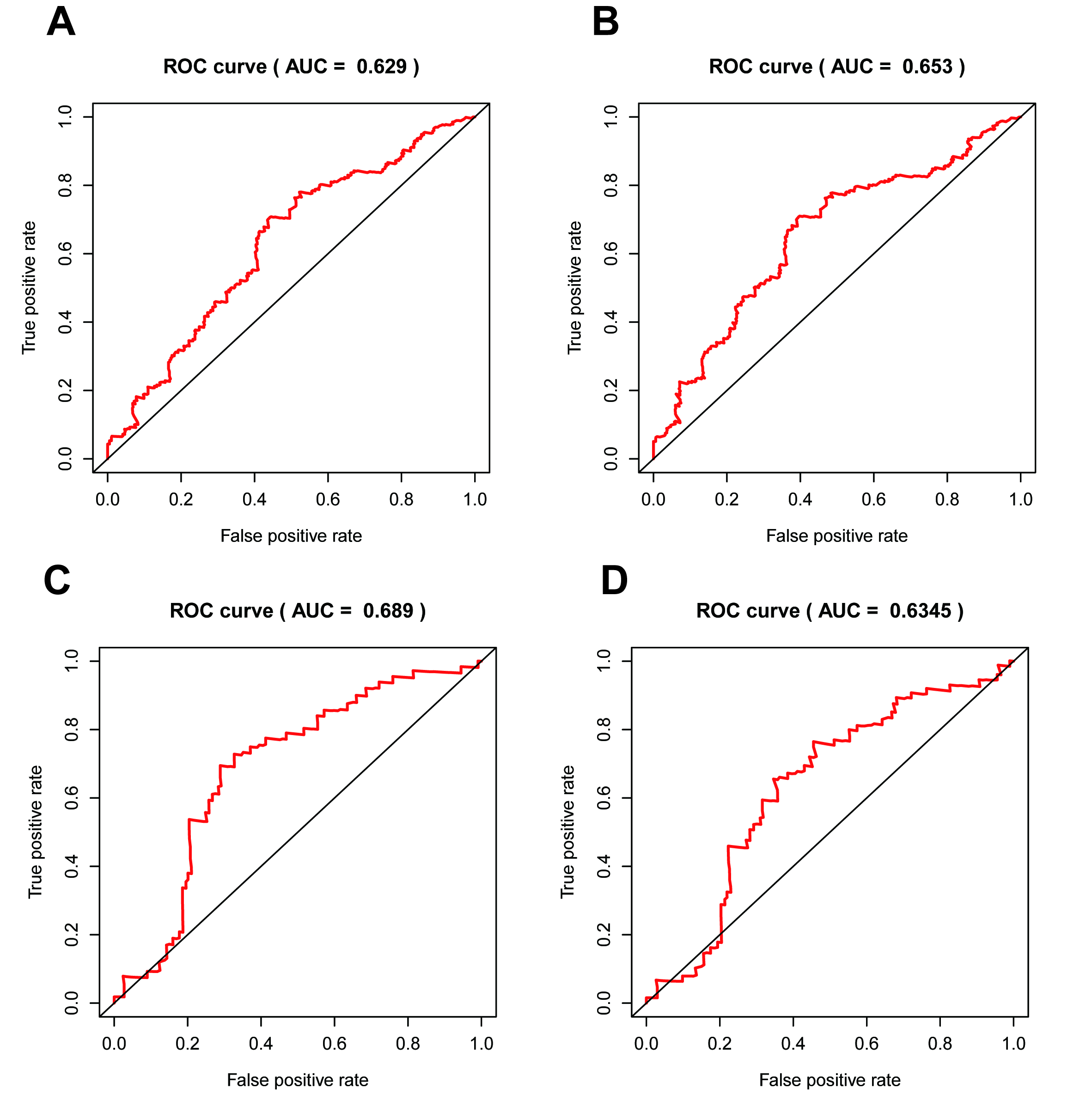

Supplement: Supplementary file 3 — Additional file 3: Figure S3. Time-dependent ROC analysis in the TCGA cohort and GSE13507 cohort. (A-B) The area under the curve (AUC) at 3-year (A) and at 5-year (B) in the TCGA cohort. (C-D) AUC at 3-year (C) and at 5-year (D) in the GSE13507 cohort. [file 12935_2021_2096_MOESM3_ESM.tif]

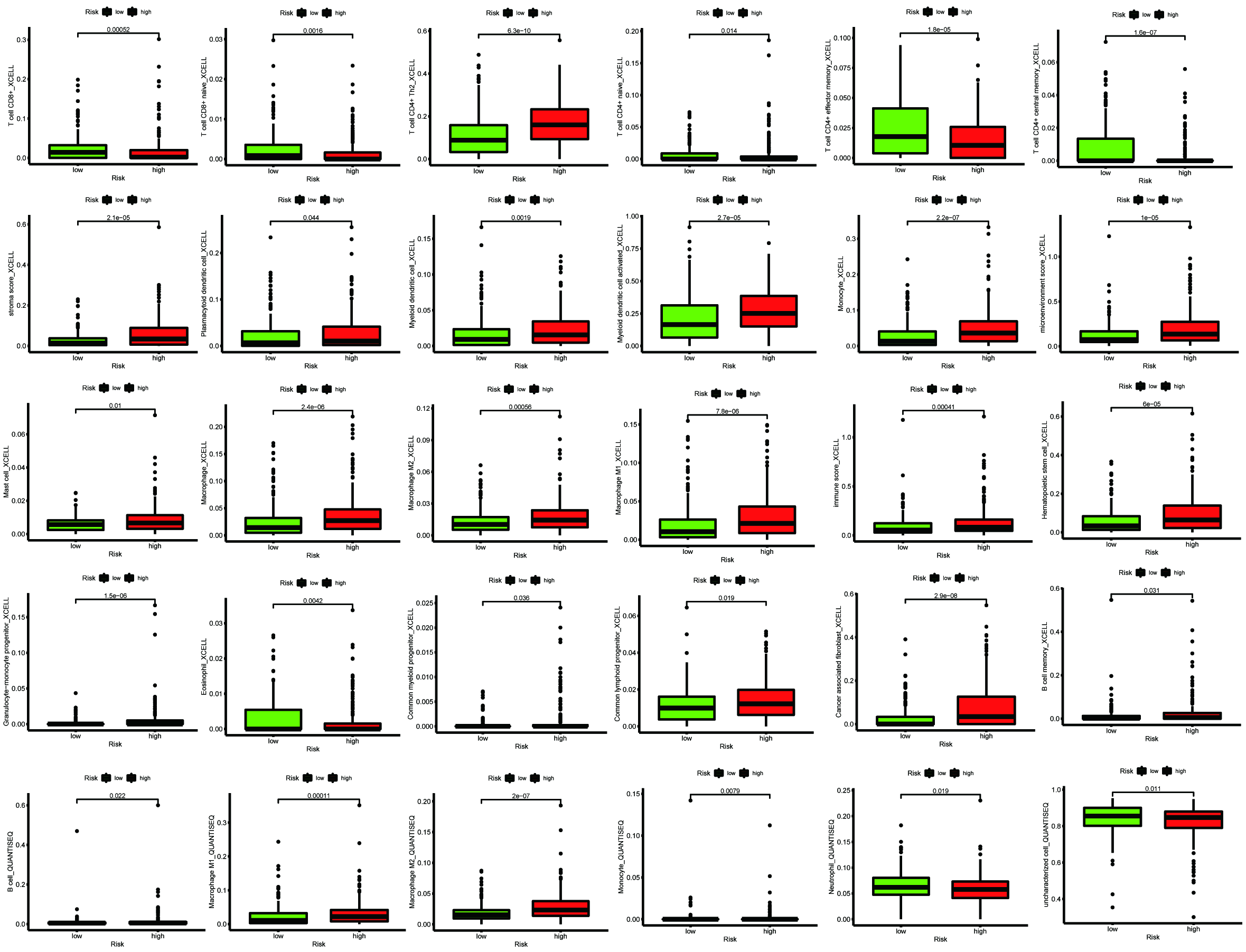

Supplement: Supplementary file 4 — Additional file 4: Figure S4. The representative results of the evaluation of tumor infiltrating immune cells with the FAGs signature (riskScore) model based on XCELL and QUANTISEQ algorithms. [file 12935_2021_2096_MOESM4_ESM.tif]

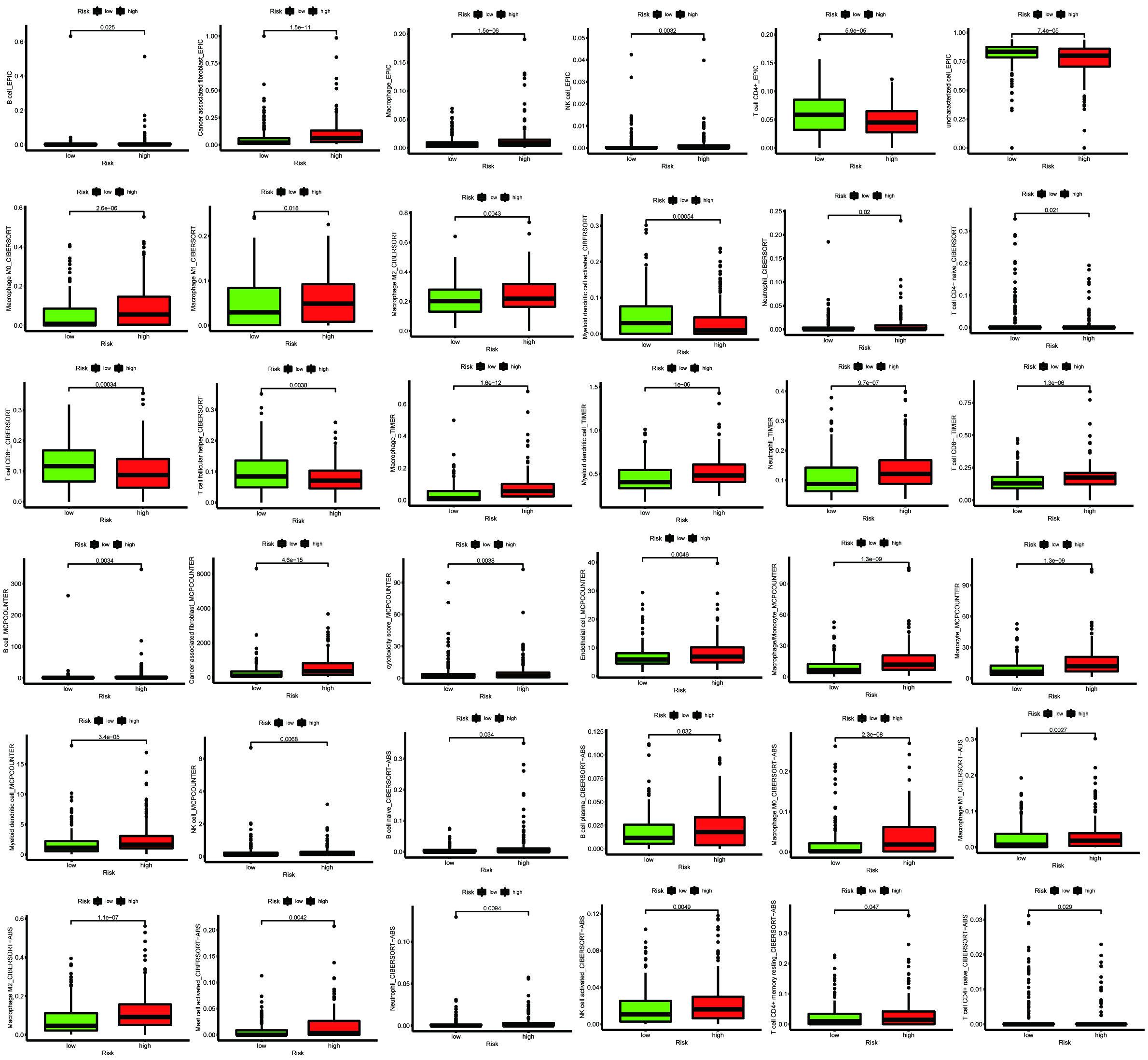

Supplement: Supplementary file 5 — Additional file 5: Figure S5. The representative results of the evaluation of tumor infiltrating immune cells with the FAGs signature (riskScore) based on TIMER, MCPCOUNT, EPIC, CIBERSORT and CIBERSORT-ABS algorithms. [file 12935_2021_2096_MOESM5_ESM.tif]

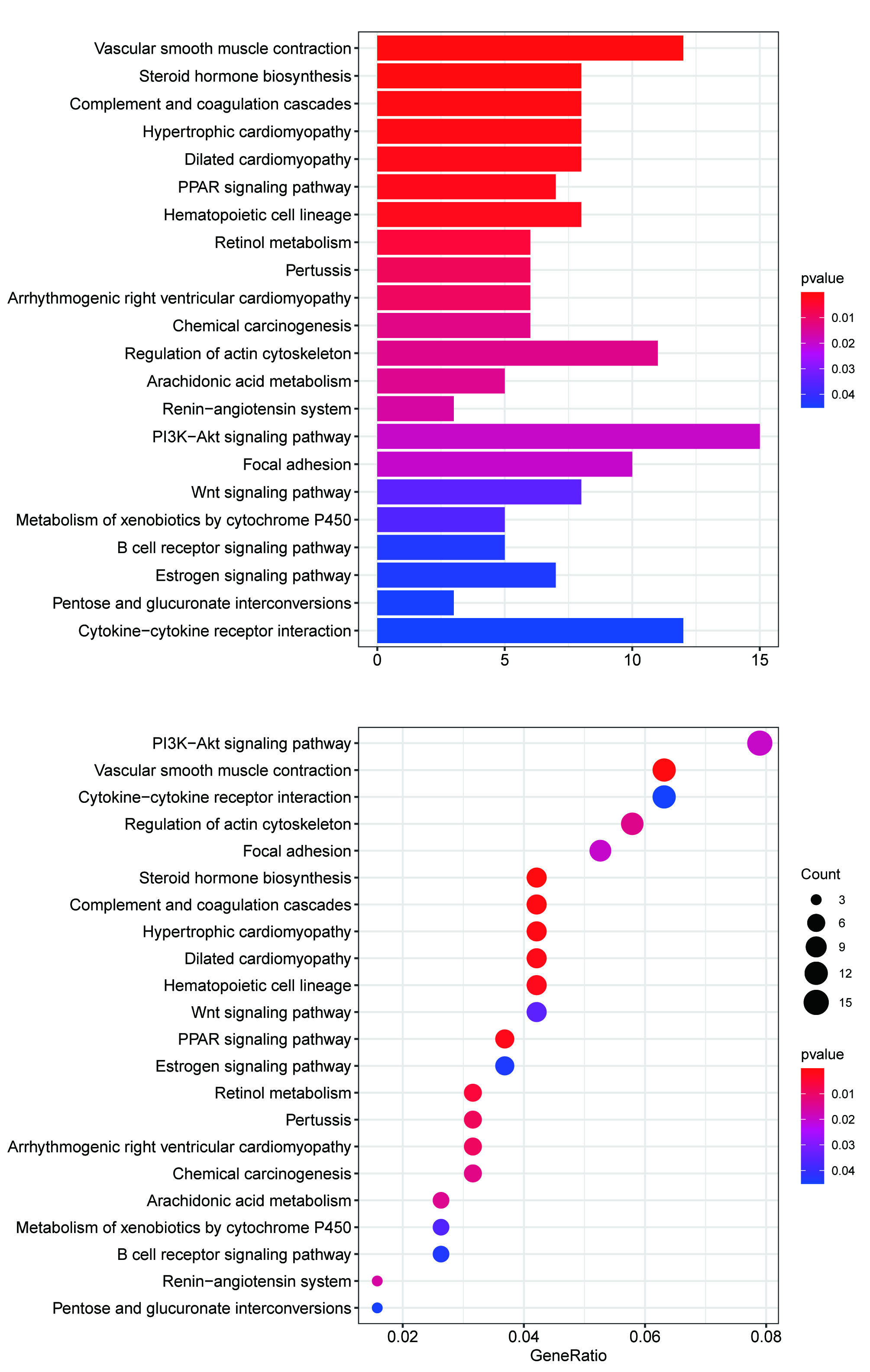

Supplement: Supplementary file 6 — Additional file 6: Figure S6. GO enrichment analysis of differently expressed ferroptosis-associated genes (DEFAGs) between two risk groups. [file 12935_2021_2096_MOESM6_ESM.tif]

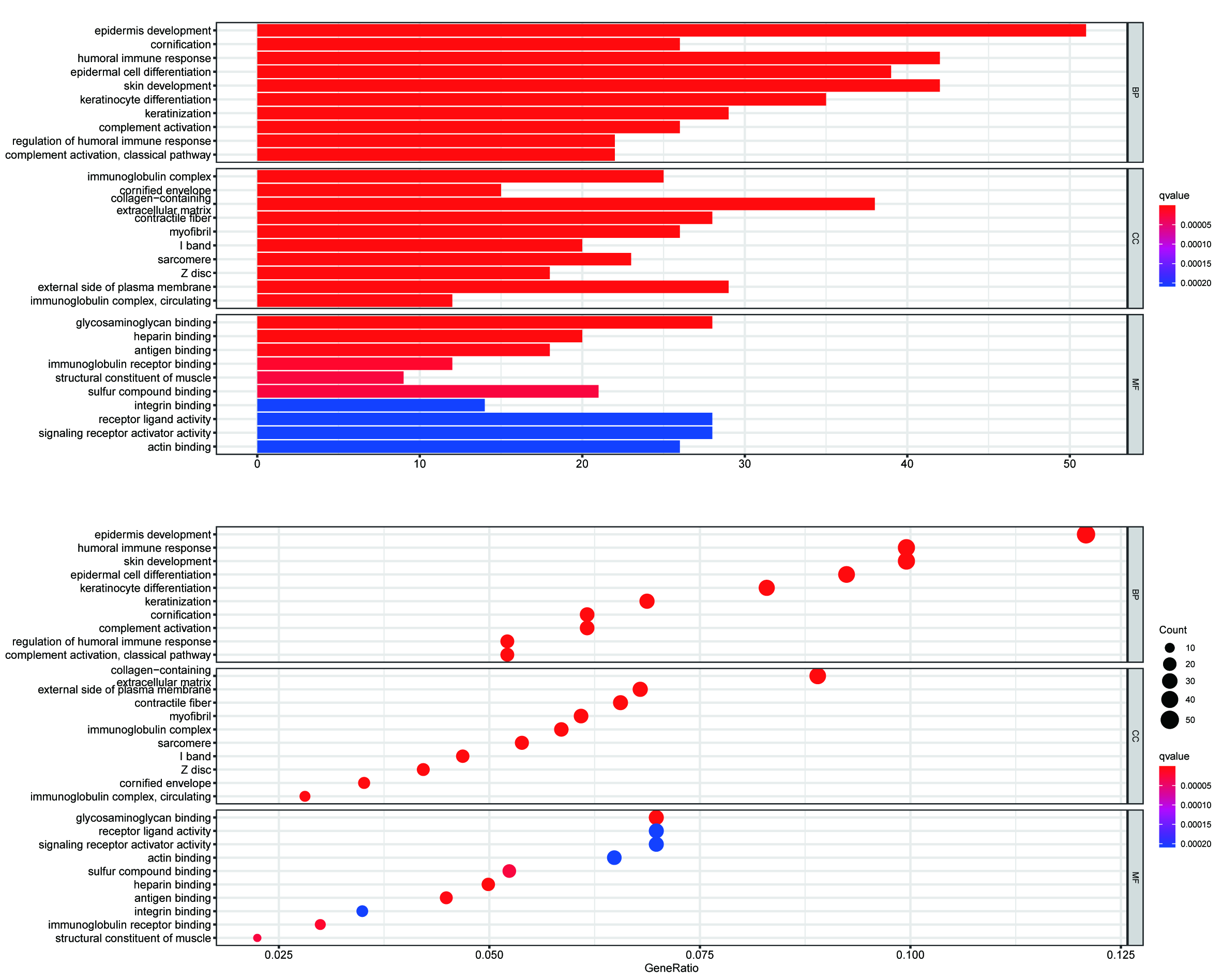

Supplement: Supplementary file 7 — Additional file 7: Figure S7. KEGG enrichment analysis of differently expressed ferroptosis-associated genes (DEFAGs) between two risk groups. [file 12935_2021_2096_MOESM7_ESM.tif]

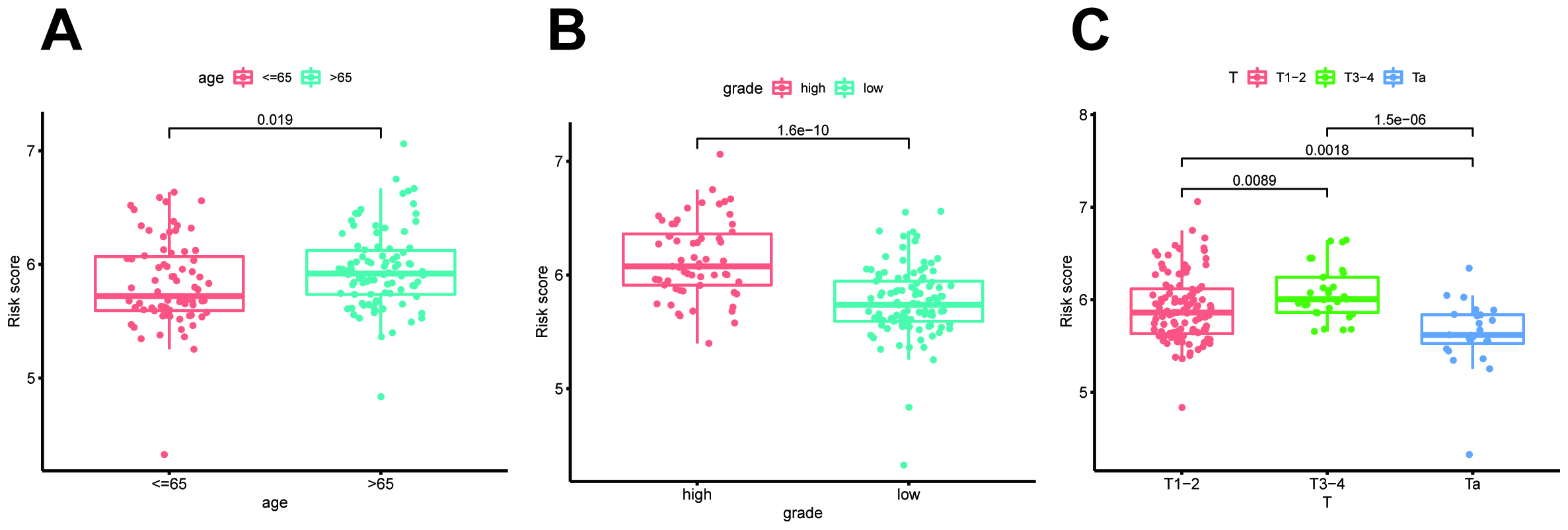

Supplement: Supplementary file 8 — Additional file 8: Figure S8. The scatter diagram indicated that age (A), grade (B) and stage T (C) were significantly associated with the riskScore. [file 12935_2021_2096_MOESM8_ESM.tif]

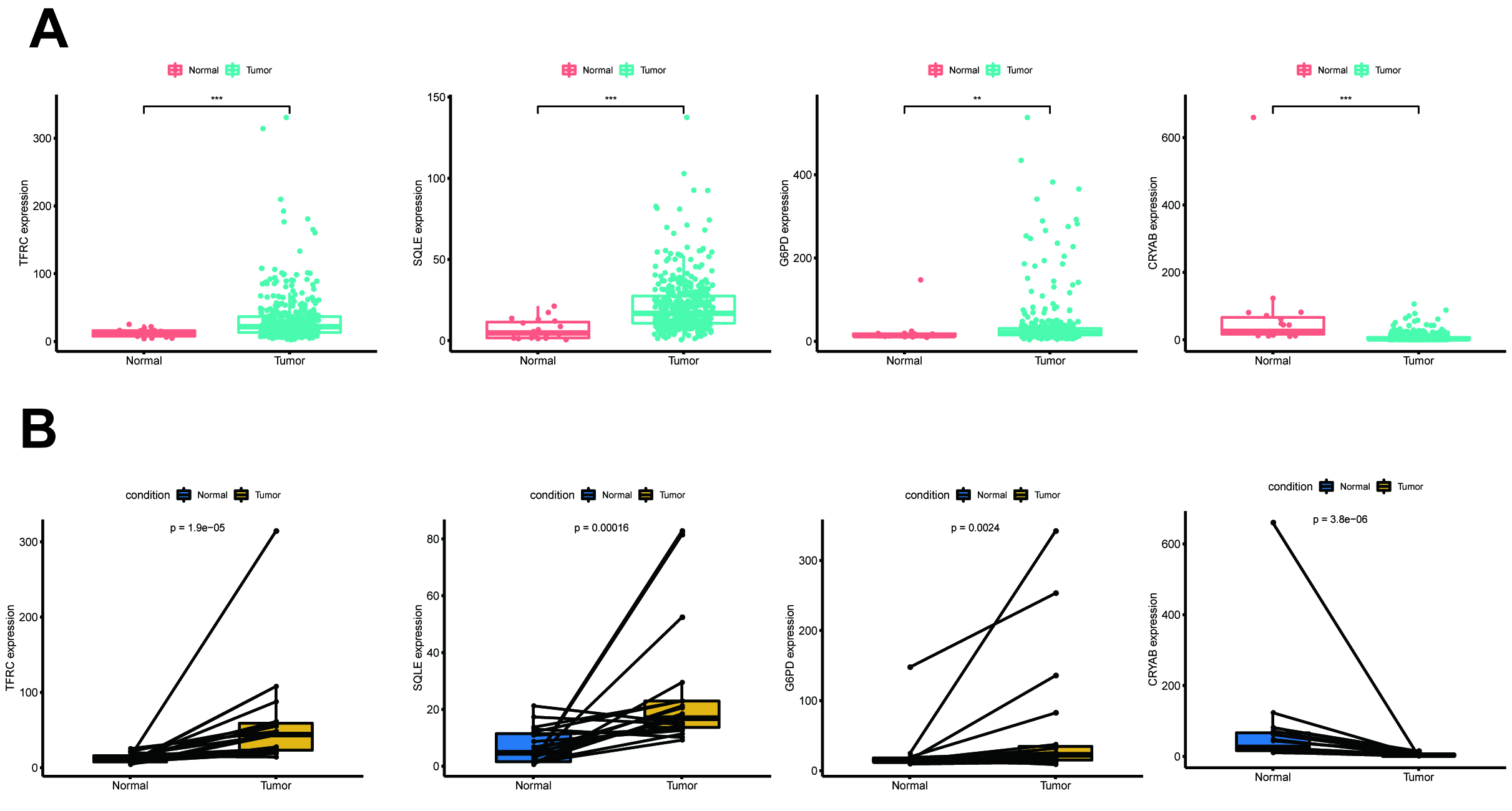

Supplement: Supplementary file 9 — Additional file 9: Figure S9. The expression levels of four hub FAGs (CRYAB, TFRC, SQLE and G6PD) in the signature. (A) mRNA expression in total BLCA and normal tissues; (B) mRNA expression in BLCA and paired bladder tissues. ***, P < 0.001; **, P < 0.01. [file 12935_2021_2096_MOESM9_ESM.tif]

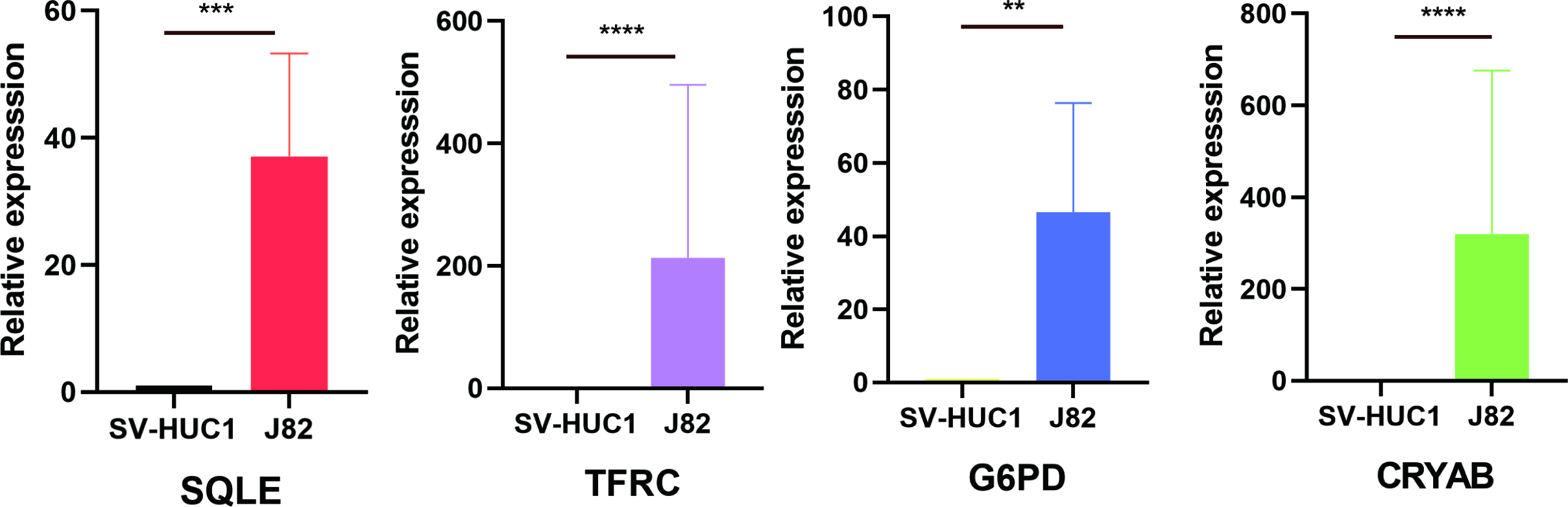

Supplement: Supplementary file 10 — Additional file 10: Figure S10. The expression levels of four hub FAGs (CRYAB, TFRC, SQLE and G6PD) were verified by setting β-actin as an internal control in the QRT-PCR experiment. ***, P < 0.001; **, P < 0.01. [file 12935_2021_2096_MOESM10_ESM.tif]
