# Supplementary material for: A novel signature constructed by ferroptosis-associated genes (FAGs) for the prediction of prognosis in bladder urothelial carcinoma (BLCA) and associated with immune infiltration
Source: Cancer Cell Int. 2021 Aug 6;21:414. doi: 10.1186/s12935-021-02096-3 (PMC8349026; doi:10.1186/s12935-021-02096-3)
Supplement: Supplementary file 11 — Additional file 11: Table S1. A total of 60 ferroptosis-associated genes (FAGs). [file 12935_2021_2096_MOESM11_ESM.docx]

Additional file 11: Table S1. A total of 60 ferroptosis-associated genes (FAGs).

ACSL4

AKR1C1

AKR1C2

AKR1C3

ALOX15

ALOX5

ALOX12

ATP5MC3

CARS1

CBS

CD44

CHAC1

CISD1

CS

DPP4

FANCD2

GCLC

GCLM

GLS2

GPX4

GSS

HMGCR

HSPB1

CRYAB

LPCAT3

MT1G

NCOA4

PTGS2

RPL8

SAT1

SLC7A11

FDFT1

TFRC

TP53

EMC2

AIFM2

PHKG2

HSBP1

ACO1

FTH1

STEAP3

NFS1

ACSL3

ACACA

PEBP1

ZEB1

SQLE

FADS2

NFE2L2

KEAP1

NQO1

NOX1

ABCC1

SLC1A5

GOT1

G6PD

PGD

IREB2

HMOX1

ACSF2
