# Supplementary material for: A novel signature constructed by ferroptosis-associated genes (FAGs) for the prediction of prognosis in bladder urothelial carcinoma (BLCA) and associated with immune infiltration
Source: Cancer Cell Int. 2021 Aug 6;21:414. doi: 10.1186/s12935-021-02096-3 (PMC8349026; doi:10.1186/s12935-021-02096-3)
Supplement: Supplementary file 12 — Additional file 12: Table S2. Clinical characteristics of BLCA patients in the TCGA cohort. [file 12935_2021_2096_MOESM12_ESM.docx]

Additional file 12: Table S2. Clinical characteristics of BLCA patients in the TCGA cohort.

| characteristics | TCGA cohort (414) | % |
| --- | --- | --- |
| Age  Gender  Male  Female  Grade  High grade  Low grade  unknown  Stage  Stage I  Stage II  Stage III  Stage IV  unknown | 69(34-90)  305  109  388  21  5  2  131  141  136  4 | 73.67  26.33  93.72  5.07  1.21  0.48  31.64  34.06  32.85  0.97 |
