# Supplementary material for: A novel signature constructed by ferroptosis-associated genes (FAGs) for the prediction of prognosis in bladder urothelial carcinoma (BLCA) and associated with immune infiltration
Source: Cancer Cell Int. 2021 Aug 6;21:414. doi: 10.1186/s12935-021-02096-3 (PMC8349026; doi:10.1186/s12935-021-02096-3)
Supplement: Supplementary file 13 — Additional file 13: Table S3. GO analysis of differently expressed ferroptosis -related genes. [file 12935_2021_2096_MOESM13_ESM.docx]

Additional file 13: Table S3. GO analysis of differently expressed ferroptosis -related genes.

| ID | Term P value |  |
| --- | --- | --- |
| \| GO:0006749 \| \| --- \| \| GO:0006979 \| \| GO:0006750 \| \| GO:0019184 \| \| GO:0007568 \| \| GO:0055076 \| \| GO:0010038 \| \| GO:0006790 \| \| GO:0055072 \| \| GO:0006575 \| \| GO:0031667 \| \| GO:0046916 \| \| GO:0042398 \| \| GO:0098754 \| \| GO:0006879 \| \| GO:0051188 \| \| GO:0046677 \| \| GO:0071248 \| \| GO:0035690 \| \| GO:0098869 \| \| GO:0010039 \| \| GO:0071241 \| \| GO:0007584 \| \| GO:1990748 \| \| GO:0006732 \| \| GO:0097237 \| \| GO:0032355 \| \| GO:0034605 \| \| GO:0072593 \| \| GO:0035296 \| \| GO:0050880 \| \| GO:0097746 \| \| GO:0035150 \| \| GO:0042542 \| \| GO:0034599 \| \| GO:0010656 \| \| GO:0046686 \| \| GO:0043619 \| \| GO:0003018 \| \| GO:0009408 \| \| GO:0001101 \| \| GO:0044272 \| \| GO:0006695 \| \| GO:0006520 \| \| GO:2000377 \| \| GO:1902653 \| \| GO:0043536 \| \| GO:0006098 \| \| GO:0019321 \| \| GO:0016126 \| \| GO:0006733 \| \| GO:0019372 \| \| GO:0043523 \| \| GO:2001234 \| \| GO:0000302 \| \| GO:0010660 \| \| GO:0051402 \| \| GO:0010657 \| \| GO:0019682 \| \| GO:0071498 \| \| GO:0009266 \| \| GO:0048661 \| \| GO:0010499 \| \| GO:0062012 \| \| GO:0003012 \| \| GO:0043200 \| \| GO:0019430 \| \| GO:0051156 \| \| GO:0071450 \| \| GO:0071451 \| \| GO:1990776 \| \| GO:0045471 \| \| GO:0000303 \| \| GO:0071280 \| \| GO:0097193 \| \| GO:0010595 \| \| GO:0000305 \| \| GO:0042759 \| \| GO:0006739 \| \| GO:1901214 \| \| GO:0033572 \| \| GO:0034405 \| \| GO:0071276 \| \| GO:0090050 \| \| GO:0008203 \| \| GO:0015682 \| \| GO:0072512 \| \| GO:1902652 \| \| GO:0043535 \| \| GO:0070997 \| \| GO:0010664 \| \| GO:0000096 \| \| GO:0001666 \| \| GO:0016125 \| \| GO:0031331 \| \| GO:0046688 \| \| GO:0048660 \| \| GO:0044275 \| \| GO:0010634 \| \| GO:0048659 \| \| GO:0036293 \| \| GO:1904036 \| \| GO:0046165 \| \| GO:0043534 \| \| GO:0045540 \| \| GO:0106118 \| \| GO:0070482 \| \| GO:0019362 \| \| GO:0046496 \| \| GO:2001233 \| \| GO:0072524 \| \| GO:0006694 \| \| GO:0046890 \| \| GO:0010332 \| \| GO:0010665 \| \| GO:0009746 \| \| GO:0045766 \| \| GO:0009896 \| \| GO:0010662 \| \| GO:0034284 \| \| GO:0071456 \| \| GO:0010659 \| \| GO:0010658 \| \| GO:0016051 \| \| GO:0090181 \| \| GO:0036294 \| \| GO:0006826 \| \| GO:1903672 \| \| GO:2000378 \| \| GO:0009314 \| \| GO:0051187 \| \| GO:0032387 \| \| GO:0071230 \| \| GO:0010594 \| \| GO:0009743 \| \| GO:1904018 \| \| GO:0097305 \| \| GO:0071453 \| \| GO:0031669 \| \| GO:0006801 \| \| GO:0033002 \| \| GO:0006081 \| \| GO:0031670 \| \| GO:0006809 \| \| GO:0042180 \| \| GO:0045926 \| \| GO:1900034 \| \| GO:0050661 \| \| GO:0016874 \| \| GO:0050662 \| \| GO:0016881 \| \| GO:0016209 \| \| GO:0016616 \| \| GO:0016614 \| \| GO:0016705 \| \| GO:0016879 \| \| GO:0004601 \| \| GO:0016684 \| \| GO:0051536 \| \| GO:0051540 \| | \| glutathione metabolic process \| 1.98E-10 \| \| --- \| --- \| \| response to oxidative stress \| 5.45E-10 \| \| glutathione biosynthetic process \| 9.50E-09 \| \| nonribosomal peptide biosynthetic process \| 1.54E-08 \| \| aging \| 1.61E-08 \| \| transition metal ion homeostasis \| 3.53E-08 \| \| response to metal ion \| 4.27E-08 \| \| sulfur compound metabolic process \| 5.06E-08 \| \| iron ion homeostasis \| 1.09E-07 \| \| cellular modified amino acid metabolic process \| 4.60E-07 \| \| response to nutrient levels \| 4.78E-07 \| \| cellular transition metal ion homeostasis \| 5.22E-07 \| \| cellular modified amino acid biosynthetic process \| 8.89E-07 \| \| detoxification \| 1.36E-06 \| \| cellular iron ion homeostasis \| 1.99E-06 \| \| cofactor biosynthetic process \| 7.37E-06 \| \| response to antibiotic \| 7.50E-06 \| \| cellular response to metal ion \| 8.41E-06 \| \| cellular response to drug \| 1.49E-05 \| \| cellular oxidant detoxification \| 1.55E-05 \| \| response to iron ion \| 1.60E-05 \| \| cellular response to inorganic substance \| 1.60E-05 \| \| response to nutrient \| 1.67E-05 \| \| cellular detoxification \| 2.25E-05 \| \| coenzyme metabolic process \| 2.45E-05 \| \| cellular response to toxic substance \| 2.98E-05 \| \| response to estradiol \| 4.54E-05 \| \| cellular response to heat \| 4.95E-05 \| \| reactive oxygen species metabolic process \| 5.80E-05 \| \| regulation of tube diameter \| 5.86E-05 \| \| regulation of blood vessel size \| 5.86E-05 \| \| regulation of blood vessel diameter \| 5.86E-05 \| \| regulation of tube size \| 6.02E-05 \| \| response to hydrogen peroxide \| 6.35E-05 \| \| cellular response to oxidative stress \| 7.76E-05 \| \| negative regulation of muscle cell apoptotic process \| 9.28E-05 \| \| response to cadmium ion \| 0.00011 \| \| regulation of transcription from RNA polymerase II promoter in response to oxidative stress \| 0.00012 \| \| vascular process in circulatory system \| 0.00012 \| \| response to heat \| 0.00013 \| \| response to acid chemical \| 0.00014 \| \| sulfur compound biosynthetic process \| 0.00018 \| \| cholesterol biosynthetic process \| 0.00019 \| \| cellular amino acid metabolic process \| 0.00019 \| \| regulation of reactive oxygen species metabolic process \| 0.00019 \| \| secondary alcohol biosynthetic process \| 0.0002 \| \| positive regulation of blood vessel endothelial cell migration \| 0.00022 \| \| pentose-phosphate shunt \| 0.00023 \| \| pentose metabolic process \| 0.00023 \| \| sterol biosynthetic process \| 0.00024 \| \| oxidoreduction coenzyme metabolic process \| 0.00024 \| \| lipoxygenase pathway \| 0.00026 \| \| regulation of neuron apoptotic process \| 0.00026 \| \| negative regulation of apoptotic signaling pathway \| 0.00036 \| \| response to reactive oxygen species \| 0.00038 \| \| regulation of muscle cell apoptotic process \| 0.00039 \| \| neuron apoptotic process \| 0.00042 \| \| muscle cell apoptotic process \| 0.00044 \| \| glyceraldehyde-3-phosphate metabolic process \| 0.00045 \| \| cellular response to fluid shear stress \| 0.00045 \| \| response to temperature stimulus \| 0.00045 \| \| positive regulation of smooth muscle cell proliferation \| 0.00046 \| \| proteasomal ubiquitin-independent protein catabolic process \| 0.00054 \| \| regulation of small molecule metabolic process \| 0.00054 \| \| muscle system process \| 0.00057 \| \| response to amino acid \| 0.00063 \| \| removal of superoxide radicals \| 0.00064 \| \| glucose 6-phosphate metabolic process \| 0.00064 \| \| cellular response to oxygen radical \| 0.00074 \| \| cellular response to superoxide \| 0.00074 \| \| response to angiotensin \| 0.0008 \| \| response to ethanol \| 0.00085 \| \| response to superoxide \| 0.00086 \| \| cellular response to copper ion \| 0.00086 \| \| intrinsic apoptotic signaling pathway \| 0.00086 \| \| positive regulation of endothelial cell migration \| 0.00091 \| \| response to oxygen radical \| 0.00092 \| \| long-chain fatty acid biosynthetic process \| 0.00092 \| \| NADP metabolic process \| 0.00105 \| \| regulation of neuron death \| 0.00115 \| \| transferrin transport \| 0.00132 \| \| response to fluid shear stress \| 0.0014 \| \| cellular response to cadmium ion \| 0.0014 \| \| positive regulation of cell migration involved in sprouting angiogenesis \| 0.0014 \| \| cholesterol metabolic process \| 0.00144 \| \| ferric iron transport \| 0.00147 \| \| trivalent inorganic cation transport \| 0.00147 \| \| secondary alcohol metabolic process \| 0.00158 \| \| regulation of blood vessel endothelial cell migration \| 0.00161 \| \| neuron death \| 0.00171 \| \| negative regulation of striated muscle cell apoptotic process \| 0.00172 \| \| sulfur amino acid metabolic process \| 0.00189 \| \| response to hypoxia \| 0.00191 \| \| sterol metabolic process \| 0.00192 \| \| positive regulation of cellular catabolic process \| 0.00195 \| \| response to copper ion \| 0.00197 \| \| regulation of smooth muscle cell proliferation \| 0.00202 \| \| cellular carbohydrate catabolic process \| 0.00206 \| \| positive regulation of epithelial cell migration \| 0.00209 \| \| smooth muscle cell proliferation \| 0.00209 \| \| response to decreased oxygen levels \| 0.00213 \| \| negative regulation of epithelial cell apoptotic process \| 0.00216 \| \| alcohol biosynthetic process \| 0.00223 \| \| blood vessel endothelial cell migration \| 0.00242 \| \| regulation of cholesterol biosynthetic process \| 0.00254 \| \| regulation of sterol biosynthetic process \| 0.00254 \| \| response to oxygen levels \| 0.00268 \| \| pyridine nucleotide metabolic process \| 0.00278 \| \| nicotinamide nucleotide metabolic process \| 0.00278 \| \| regulation of apoptotic signaling pathway \| 0.00298 \| \| pyridine-containing compound metabolic process \| 0.00303 \| \| steroid biosynthetic process \| 0.00308 \| \| regulation of lipid biosynthetic process \| 0.00317 \| \| response to gamma radiation \| 0.00318 \| \| regulation of cardiac muscle cell apoptotic process \| 0.00329 \| \| response to hexose \| 0.00335 \| \| positive regulation of angiogenesis \| 0.00344 \| \| positive regulation of catabolic process \| 0.00346 \| \| regulation of striated muscle cell apoptotic process \| 0.00352 \| \| response to monosaccharide \| 0.00359 \| \| cellular response to hypoxia \| 0.00359 \| \| cardiac muscle cell apoptotic process \| 0.00364 \| \| striated muscle cell apoptotic process \| 0.00388 \| \| carbohydrate biosynthetic process \| 0.00394 \| \| regulation of cholesterol metabolic process \| 0.004 \| \| cellular response to decreased oxygen levels \| 0.00409 \| \| iron ion transport \| 0.00413 \| \| positive regulation of sprouting angiogenesis \| 0.00413 \| \| negative regulation of reactive oxygen species metabolic process \| 0.00413 \| \| response to radiation \| 0.00424 \| \| cofactor catabolic process \| 0.00426 \| \| negative regulation of intracellular transport \| 0.00452 \| \| cellular response to amino acid stimulus \| 0.00465 \| \| regulation of endothelial cell migration \| 0.00476 \| \| response to carbohydrate \| 0.00482 \| \| positive regulation of vasculature development \| 0.00482 \| \| response to alcohol \| 0.00499 \| \| cellular response to oxygen levels \| 0.00505 \| \| cellular response to nutrient levels \| 0.00523 \| \| superoxide metabolic process \| 0.00534 \| \| muscle cell proliferation \| 0.00536 \| \| cellular aldehyde metabolic process \| 0.00548 \| \| cellular response to nutrient \| 0.00548 \| \| nitric oxide biosynthetic process \| 0.00592 \| \| cellular ketone metabolic process \| 0.00594 \| \| negative regulation of growth \| 0.006 \| \| regulation of cellular response to heat \| 0.00622 \| \| NADP binding \| 7.44E-05 \| \| ligase activity \| 7.60E-05 \| \| coenzyme binding \| 8.37E-05 \| \| acid-amino acid ligase activity \| 0.00029 \| \| antioxidant activity \| 0.00033 \| \| oxidoreductase activity, acting on the CH-OH group of donors, NAD or NADP as acceptor \| 0.00086 \| \| oxidoreductase activity, acting on CH-OH group of donors \| 0.00106 \| \| oxidoreductase activity, acting on paired donors, with incorporation or reduction of molecular oxygen \| 0.00198 \| \| ligase activity, forming carbon-nitrogen bonds \| 0.00219 \| \| peroxidase activity \| 0.00305 \| \| oxidoreductase activity, acting on peroxide as acceptor \| 0.00353 \| \| iron-sulfur cluster binding \| 0.00444 \| \| metal cluster binding \| 0.00444 \| |  |
