# Supplementary material for: A novel signature constructed by ferroptosis-associated genes (FAGs) for the prediction of prognosis in bladder urothelial carcinoma (BLCA) and associated with immune infiltration
Source: Cancer Cell Int. 2021 Aug 6;21:414. doi: 10.1186/s12935-021-02096-3 (PMC8349026; doi:10.1186/s12935-021-02096-3)
Supplement: Supplementary file 14 — Additional file 14: Table S4. KEGG analysis of differently expressed ferroptosis-associated genes. [file 12935_2021_2096_MOESM14_ESM.docx]

Additional file 14: Table S4. KEGG analysis of differently expressed ferroptosis-associated genes.

| Category | ID Term | P value |
| --- | --- | --- |
| KEGG pathway  KEGG pathway  KEGG pathway  KEGG pathway  KEGG pathway  KEGG pathway  KEGG pathway | \| hsa04216 \| Ferroptosis \| \| --- \| --- \| \| hsa00480 \| Glutathione metabolism \| \| hsa05418 \| Fluid shear stress and atherosclerosis \| \| hsa01240 \| Biosynthesis of cofactors \| \| hsa05225 \| Hepatocellular carcinoma \| \| hsa00030 \| Pentose phosphate pathway \| \| hsa01200 \| Carbon metabolism \| | \| 2.26E-11 \| \| --- \| \| 1.50E-08 \| \| 0.000799 \| \| 0.001229 \| \| 0.001616 \| \| 0.003785 \| \| 0.005511 \| |
