# Supplementary material for: A novel signature constructed by ferroptosis-associated genes (FAGs) for the prediction of prognosis in bladder urothelial carcinoma (BLCA) and associated with immune infiltration
Source: Cancer Cell Int. 2021 Aug 6;21:414. doi: 10.1186/s12935-021-02096-3 (PMC8349026; doi:10.1186/s12935-021-02096-3)
Supplement: Supplementary file 15 — Additional file 15: Table S5. The individual HR and P value of ferroptosis-associated genes (FAGs) in the TCGA dataset according to the univariate Cox analysis. [file 12935_2021_2096_MOESM15_ESM.docx]

Additional file 15: Table S5. The individual HR and P value of ferroptosis-associated genes (FAGs) in the TCGA dataset according to the univariate Cox analysis.

| id | HR | HR.95L | HR.95H | P value |
| --- | --- | --- | --- | --- |
| GCLM  CRYAB  TFRC  ZEB1  SQLE  G6PD  PGD  ACSF2 | \| 1.214333 \| \| --- \| \| 1.16579 \| \| 1.173374 \| \| 1.230669 \| \| 1.198022 \| \| 1.216691 \| \| 1.181185 \| \| 0.849285 \| | \| 1.062891 \| \| --- \| \| 1.057259 \| \| 1.026955 \| \| 1.031634 \| \| 1.024551 \| \| 1.067057 \| \| 1.015204 \| \| 0.745046 \| | \| 1.387353 \| \| --- \| \| 1.285461 \| \| 1.340669 \| \| 1.468104 \| \| 1.400864 \| \| 1.387308 \| \| 1.374303 \| \| 0.968109 \| | \| 0.004271 \| \| --- \| \| 0.002093 \| \| 0.018719 \| \| 0.021112 \| \| 0.023581 \| \| 0.003397 \| \| 0.031141 \| \| 0.014482 \| |
