# Supplementary material for: A novel signature constructed by ferroptosis-associated genes (FAGs) for the prediction of prognosis in bladder urothelial carcinoma (BLCA) and associated with immune infiltration
Source: Cancer Cell Int. 2021 Aug 6;21:414. doi: 10.1186/s12935-021-02096-3 (PMC8349026; doi:10.1186/s12935-021-02096-3)
Supplement: Supplementary file 16 — Additional file 16: Table S6. The regression coefficients and HR of four ferroptosis-associated genes (FAGs) according to the multiple stepwise Cox regression analysis. [file 12935_2021_2096_MOESM16_ESM.docx]

Additional file 16: Table S6. The regression coefficients and HR of four ferroptosis-associated genes (FAGs) according to the multiple stepwise Cox regression analysis.

| ID | Coefficient | HR |
| --- | --- | --- |
| \| CRYAB \| \| --- \| \| TFRC \| \| SQLE \| \| G6PD \| | \| 0.18208 \| \| --- \| \| 0.12253 \| \| 0.134168 \| \| 0.180591 \| | \| 1.19971 \| \| --- \| \| 1.130353 \| \| 1.143585 \| \| 1.197925 \| |
