# Supplementary material for: A novel signature constructed by ferroptosis-associated genes (FAGs) for the prediction of prognosis in bladder urothelial carcinoma (BLCA) and associated with immune infiltration
Source: Cancer Cell Int. 2021 Aug 6;21:414. doi: 10.1186/s12935-021-02096-3 (PMC8349026; doi:10.1186/s12935-021-02096-3)
Supplement: Supplementary file 17 — Additional file 17: Table S7. The detail comparison results of the correlation between tumor-infiltrating immune cells and riskScore. [file 12935_2021_2096_MOESM17_ESM.docx]

Additional file 17: Table S7. The detail comparison results of correlation ship between tumor-infiltrating immune cells and riskScore.

| Symbol | Correction | P value |
| --- | --- | --- |
| \| T cell CD4+_TIMERc \| \| --- \| \| T cell CD8+_TIMER \| \| Neutrophil TIMER \| \| Macrophage TIMER \| \| Myeloid dendritic cell TIMER \| \| B cell naïve CIBERSORT \| \| T cell CD8+ CIBERSORT \| \| T cell CD4+ naïve CIBERSORT \| \| T cell follicular helper CIBERSORT \| \| NK cell resting CIBERSORT \| \| Macrophage M0 CIBERSORT \| \| Macrophage M1 CIBERSORT \| \| Macrophage M2 CIBERSORT \| \| Myeloid dendritic cell activated CIBERSORT \| \| Neutrophil CIBERSORT \| \| B cell naïve CIBERSORT-ABS \| \| B cell plasma CIBERSORT-ABS \| \| T cell CD8+ CIBERSORT-ABS \| \| T cell CD4+ naïve CIBERSORT-ABS \| \| T cell CD4+ memory resting CIBERSORT-ABS \| \| T cell follicular helper CIBERSORT-ABS \| \| NK cell activated CIBERSORT-ABS \| \| Monocyte CIBERSORT-ABS \| \| Macrophage M0 CIBERSORT-ABS \| \| Macrophage M1 CIBERSORT-ABS \| \| Macrophage M2 CIBERSORT-ABS \| \| Mast cell activated CIBERSORT-ABS \| \| Neutrophil CIBERSORT-ABS \| \| B cell QUANTISEQ \| \| Macrophage M1 QUANTISEQ \| \| Macrophage M2 QUANTISEQ \| \| Monocyte QUANTISEQ \| \| T cell CD8+ QUANTISEQ \| \| T cell regulatory (Tregs) QUANTISEQ \| \| Myeloid dendritic cell QUANTISEQ \| \| uncharacterized cell QUANTISEQ \| \| T cell CD8+ MCPCOUNTER \| \| cytotoxicity score MCPCOUNTER \| \| NK cell MCPCOUNTER \| \| B cell MCPCOUNTER \| \| Monocyte MCPCOUNTER \| \| Macrophage/Monocyte MCPCOUNTER \| \| Myeloid dendritic cell MCPCOUNTER \| \| Endothelial cell MCPCOUNTER \| \| Cancer associated fibroblast MCPCOUNTER \| \| Myeloid dendritic cell activated XCELL \| \| B cell XCELL \| \| T cell CD4+ naïve XCELL \| \| T cell CD4+ central memory XCELL \| \| T cell CD4+ effector memory XCELL \| \| T cell CD8+ naïve XCELL \| \| T cell CD8+ XCELL \| \| Common lymphoid progenitor XCELL \| \| Common myeloid progenitor XCELL \| \| Myeloid dendritic cell XCELL \| \| Eosinophil XCELL \| \| Cancer associated fibroblast XCELL \| \| Granulocyte-monocyte progenitor XCELL \| \| Hematopoietic stem cell XCELL \| \| Macrophage XCELL \| \| Macrophage M1 XCELL \| \| Macrophage M2 XCELL \| \| Mast cell XCELL \| \| B cell memory XCELL \| \| Monocyte XCELL \| \| B cell naïve XCELL \| \| T cell NK XCELL \| \| Plasmacytoid dendritic cell XCELL \| \| T cell CD4+ Th1 XCELL \| \| T cell CD4+ Th2 XCELL \| \| immune score XCELL \| \| stroma score XCELL \| \| microenvironment score XCELL \| \| B cell EPIC \| \| Cancer associated fibroblast EPIC \| \| T cell CD4+ EPIC \| \| Macrophage EPIC \| \| NK cell EPIC \| \| uncharacterized cell EPIC \| | \| 0.103717878 \| \| --- \| \| 0.285499222 \| \| 0.32180858 \| \| 0.416535736 \| \| 0.305799639 \| \| 0.104971067 \| \| -0.140197646 \| \| -0.186173256 \| \| -0.154607982 \| \| -0.129891077 \| \| 0.242127768 \| \| 0.209346337 \| \| 0.149422232 \| \| -0.211252645 \| \| 0.126630351 \| \| 0.154546677 \| \| 0.144395901 \| \| 0.118191144 \| \| -0.179516975 \| \| 0.141492856 \| \| 0.141144383 \| \| 0.199134745 \| \| 0.105059648 \| \| 0.286019136 \| \| 0.245791367 \| \| 0.323449348 \| \| 0.152821991 \| \| 0.152549767 \| \| 0.159471109 \| \| 0.281383768 \| \| 0.337878684 \| \| 0.160507767 \| \| 0.124528884 \| \| 0.175708787 \| \| -0.136465251 \| \| -0.192734703 \| \| 0.149156219 \| \| 0.201999486 \| \| 0.213789112 \| \| 0.184252068 \| \| 0.386662614 \| \| 0.386662614 \| \| 0.259016809 \| \| 0.133802892 \| \| 0.461173605 \| \| 0.265887886 \| \| 0.143623544 \| \| -0.153439389 \| \| -0.290016075 \| \| -0.210353268 \| \| -0.200831905 \| \| -0.173043007 \| \| 0.145392935 \| \| 0.123100067 \| \| 0.191385714 \| \| -0.178192439 \| \| 0.315620506 \| \| 0.260356105 \| \| 0.198573551 \| \| 0.278864239 \| \| 0.282431294 \| \| 0.180197838 \| \| 0.164544333 \| \| 0.15983176 \| \| 0.287686965 \| \| 0.128282368 \| \| -0.123745777 \| \| 0.147078515 \| \| -0.116640152 \| \| 0.37917456 \| \| 0.219767071 \| \| 0.219472537 \| \| 0.260484302 \| \| 0.160057328 \| \| 0.40513021 \| \| -0.228783239 \| \| 0.301847823 \| \| 0.18597276 \| \| -0.226485378 \| | \| 0.03740915 \| \| --- \| \| 5.36E-09 \| \| 4.73E-11 \| \| 2.41E-18 \| \| 4.43E-10 \| \| 0.03515601 \| \| 0.004807689 \| \| 0.000170795 \| \| 0.001853027 \| \| 0.009040637 \| \| 8.70E-07 \| \| 2.27E-05 \| \| 0.002636552 \| \| 1.90E-05 \| \| 0.010945286 \| \| 0.001860886 \| \| 0.003672976 \| \| 0.017614388 \| \| 0.000292277 \| \| 0.004427809 \| \| 0.004527255 \| \| 5.68E-05 \| \| 0.035001211 \| \| 5.02E-09 \| \| 5.86E-07 \| \| 2.88E-11 \| \| 0.00209488 \| \| 0.002134179 \| \| 0.001318166 \| \| 9.04E-09 \| \| 3.22E-12 \| \| 0.001224337 \| \| 0.012353562 \| \| 0.000394131 \| \| 0.006072167 \| \| 0.00010165 \| \| 0.0026839 \| \| 4.57E-05 \| \| 1.57E-05 \| \| 0.000204632 \| \| 4.49E-16 \| \| 4.49E-16 \| \| 1.48E-07 \| \| 0.007185525 \| \| 0 \| \| 6.00E-08 \| \| 0.003861494 \| \| 0.002008191 \| \| 2.99E-09 \| \| 2.07E-05 \| \| 4.89E-05 \| \| 0.000484138 \| \| 0.003441956 \| \| 0.013400183 \| \| 0.00011068 \| \| 0.000324531 \| \| 9.00E-11 \| \| 1.14E-07 \| \| 5.97E-05 \| \| 1.24E-08 \| \| 7.93E-09 \| \| 0.000276886 \| \| 0.000914562 \| \| 0.001284798 \| \| 4.05E-09 \| \| 0.009939968 \| \| 0.012917896 \| \| 0.003081187 \| \| 0.019167276 \| \| 3.16E-15 \| \| 8.93E-06 \| \| 8.73E-06 \| \| 1.26E-07 \| \| 0.001280174 \| \| 0 \| \| 3.70E-06 \| \| 7.55E-10 \| \| 0.000177985 \| \| 4.65E-06 \| |
