# Supplementary material for: A novel signature constructed by ferroptosis-associated genes (FAGs) for the prediction of prognosis in bladder urothelial carcinoma (BLCA) and associated with immune infiltration
Source: Cancer Cell Int. 2021 Aug 6;21:414. doi: 10.1186/s12935-021-02096-3 (PMC8349026; doi:10.1186/s12935-021-02096-3)
Supplement: Supplementary file 18 — Additional file 18: Table S8. Clinical characteristics of BLCA patients in the GSE13507 dataset. [file 12935_2021_2096_MOESM18_ESM.docx]

Additional file 18: Table S8. Clinical characteristics of BLCA patients in the GSE13507 dataset.

| Clinical characteristics | Total (165) | % |
| --- | --- | --- |
| Age  ≤65  >65  Gender  Female  Male  Grade  High  Low  T  Ta  T1-2  T3-4  M  M0  M1  N  N0-1  N2-3  Nx | 74  91  30  135  60  105  24  111  30  158  7  157  7  1 | 44.85%  55.15%  18.18%  81.82%  36.36%  63.64%  14.55%  67.27%  18.18%  95.76%  4.24%  95.15%  4.24%  1% |
